# Identification of genetic networks that act in the somatic cells of the testis to mediate the developmental program of spermatogenesis

**DOI:** 10.1371/journal.pgen.1007026

**Published:** 2017-09-28

**Authors:** Michael John Fairchild, Fayeza Islam, Guy Tanentzapf

**Affiliations:** Department of Cellular and Physiological Sciences, University of British Columbia, Vancouver, British Columbia, Canada; NYU School of Medicine, UNITED STATES

## Abstract

Spermatogenesis is a dynamic developmental process requiring precisely timed transitions between discrete stages. Specifically, the germline undergoes three transitions: from mitotic spermatogonia to spermatocytes, from meiotic spermatocytes to spermatids, and from morphogenetic spermatids to spermatozoa. The somatic cells of the testis provide essential support to the germline throughout spermatogenesis, but their precise role during these developmental transitions has not been comprehensively explored. Here, we describe the identification and characterization of genes that are required in the somatic cells of the *Drosophila melanogaster* testis for progress through spermatogenesis. Phenotypic analysis of candidate genes pinpointed the stage of germline development disrupted. Bioinformatic analysis revealed that particular gene classes were associated with specific developmental transitions. Requirement for genes associated with endocytosis, cell polarity, and microtubule-based transport corresponded with the development of spermatogonia, spermatocytes, and spermatids, respectively. Overall, we identify mechanisms that act specifically in the somatic cells of the testis to regulate spermatogenesis.

## Author summary

Sexual reproduction in animals requires the production of male and female gametes, spermatozoa and ova, respectively. Gametes are derived from specialized cells known as the germline through a process called gametogenesis. Gametogenesis typically takes place in a gonad and requires the germ cells to be surrounded by specialized somatic cells that support germline development. While many prior studies have identified germline specific genes required for gametogenesis few have systematically identified genes required in the somatic cells for gametogenesis. To this end we performed an RNAi screen where we disrupted the function of genes specifically in the somatic cyst cells of the *Drosophila melanogaster* testis. Using fertility assays we identified 281 genes that are required in somatic cyst cells for fertility. To better understand the role of these genes in regulating spermatogenesis we classified the resulting phenotypes by the stage of germline development disrupted. This revealed distinct sets of genes required to support specific stages of spermatogenesis. Our study characterizes the somatic specific defects resulting from disruption of candidate genes and provides insight into their function in the testes. Overall, our findings reveal the mechanisms controlling *Drosophila melanogaster* spermatogenesis and provide a resource for studying soma-germline interactions more broadly.

## Introduction

Spermatogenesis is a highly choreographed developmental process that involves dramatic changes in cell morphology and the integration of multiple regulatory cues. Orchestrating such a process would be challenging even if spermatogenesis involved only one cell type, but it in fact includes two very different types of cells, somatic cells (soma) and germ cells (germline). Close association between the soma and the germline is a conserved feature of spermatogenesis in animal testes [1]. Importantly, interplay between these two tissues is critical for the normal progression of spermatogenesis and disruption of one cell type severely affects the other [2,3].

Spermatogenesis can be divided into three distinct developmental stages: the spermatogonial-stage, characterized by mitotic germ cells; the spermatocyte-stage, characterized by meiotic germ cells; and the spermatid-stage, characterized by the morphogenetic changes that form spermatozoa [1]. The spermatogonial population is sustained by germline stem cells (GSCs). When GSCs divide, the resulting daughter cells can produce either a GSC or a spermatogonial cell that proceeds through spermatogenesis. A specialized stem cell niche typically plays an instructive role in maintaining the balance between GSC self-renewal and the differentiation of spermatogonia [4,5]. During the spermatogonial-stage the germline initiates a program of differentiation and undergoes transit-amplifying mitotic divisions. These divisions are incomplete and the resulting germ cells retain cytoplasmic bridges connecting them as a ‘germ cell cyst’ [6,7]. Following the final transit-amplifying division the germline enters the spermatocyte-stage. Entry into this stage constitutes a “point-of-no-return” during spermatogenesis, as the genome is irreversibly altered and reduced from diploid to haploid by meiosis [8,9]. Progression into the spermatid-stage is marked by the completion of meiotic divisions. Spermatid development is characterized by dramatic morphological changes as the genome is compacted into a small high-density nucleus, and the cellular machinery required for fertilization and motility are formed. Finally, excess cytoplasm is removed and the intercellular bridges connecting the spermatids are severed, releasing them as individual spermatozoa [10,11].

The *Drosophila melanogaster* testis provides a powerful model to study the conserved process of spermatogenesis as it allows convenient imaging of the transitions between different stages of germline development [12–14]. The early stages of spermatogenesis occur at the closed, anterior end of the testis known as the ‘apical tip’. The apical tip of each testis holds the stem cell niche known as the hub which is made up of tightly clustered somatic cells attached to a dense accumulation of extra-cellular matrix [15]. The hub acts as a signalling centre that secretes multiple cell signalling cues to regulate the maintenance and behaviour of both germline stem cells (GSCs) and somatic cyst stem cells (CySCs). These signals include ligands for the Hedgehog (Hh) [16], Janus Kinase-Signal Transducers and Activators of Transcription (JAK-STAT) [17], and Bone Morphogenetic Protein (BMP) [18] pathways. Both GSCs and CySCs orient their centrosomes perpendicular to the hub during mitosis, resulting in predominantly asymmetrical divisions where one cell retains contact with the hub and the other is displaced and subsequently differentiates [19,20]. Displaced GSCs form gonialblasts that will go on to become spermatozoa, while displaced CySCs form cyst cells that support the germ cells throughout spermatogenesis.

A key event during the spermatogonial-stage is a process known as encapsulation whereby two cyst cells wrap each gonialblast and all three cells together form a ‘spermatocyst’. All further germ cell development takes place within the spermatocyst in the lumen formed between the two encapsulating cyst cells. Each encapsulated gonialblast proceeds through four rounds of transit-amplifying divisions resulting in two-cell, four-cell, eight-cell, and finally sixteen-cell germline cysts [21]. After reaching the sixteen-cell point, germ cells enter the spermatocyte-stage during which they undergo massive growth and expand approximately twenty-five times in volume as they move towards the basal end of the testis. The spermatocytes proceed through meiosis mid way down the testis and form sixty-four round spermatids [21]. During the spermatid-stage the germ cells polarize and elongate, their nuclei continuing to move basally while their flagellar axonemes grow to over 1800μm in length [21]. The two cyst cells encapsulating the germline also differentiate during spermatogenesis. The cyst cells undergo dramatic changes in gene expression, grow in size, and become two distinct types of cyst cells—head and tail cyst cells [3]. When the head cyst cell surrounding the spermatid nuclei reaches the end of the testis it attaches to a layer of somatic cells called the terminal epithelium. The individualized spermatozoa within the spermatocyst are then coiled at the base of the testis and threaded tail first through a narrow duct into the seminal vesicle [22]. Successful completion of spermatogenesis requires cooperation between cyst cells and germ cells. Eliminating cyst cells or disrupting their ability to encapsulate both result in the failure of germ cells to differentiate past the spermatogonial-stage [23–25].

Here we explore how the somatic cells of the testis contribute to germline development during spermatogenesis. We systematically identify genes that are required in the *Drosophila melanogaster* cyst cells for fertility. Subsequent analysis of the phenotypes resulting from somatic knockdown of candidate genes identified the stage of spermatogenesis for which their function was required. By employing bioinformatic approaches we find functional clusters of genes that mediate developmental events associated with progress through spermatogenesis. Detailed analysis of the phenotypes resulting from the disruption of several representative gene classes illustrates the diverse mechanisms through which somatic cells support the germline during spermatogenesis.

## Results

**Fig 1:**
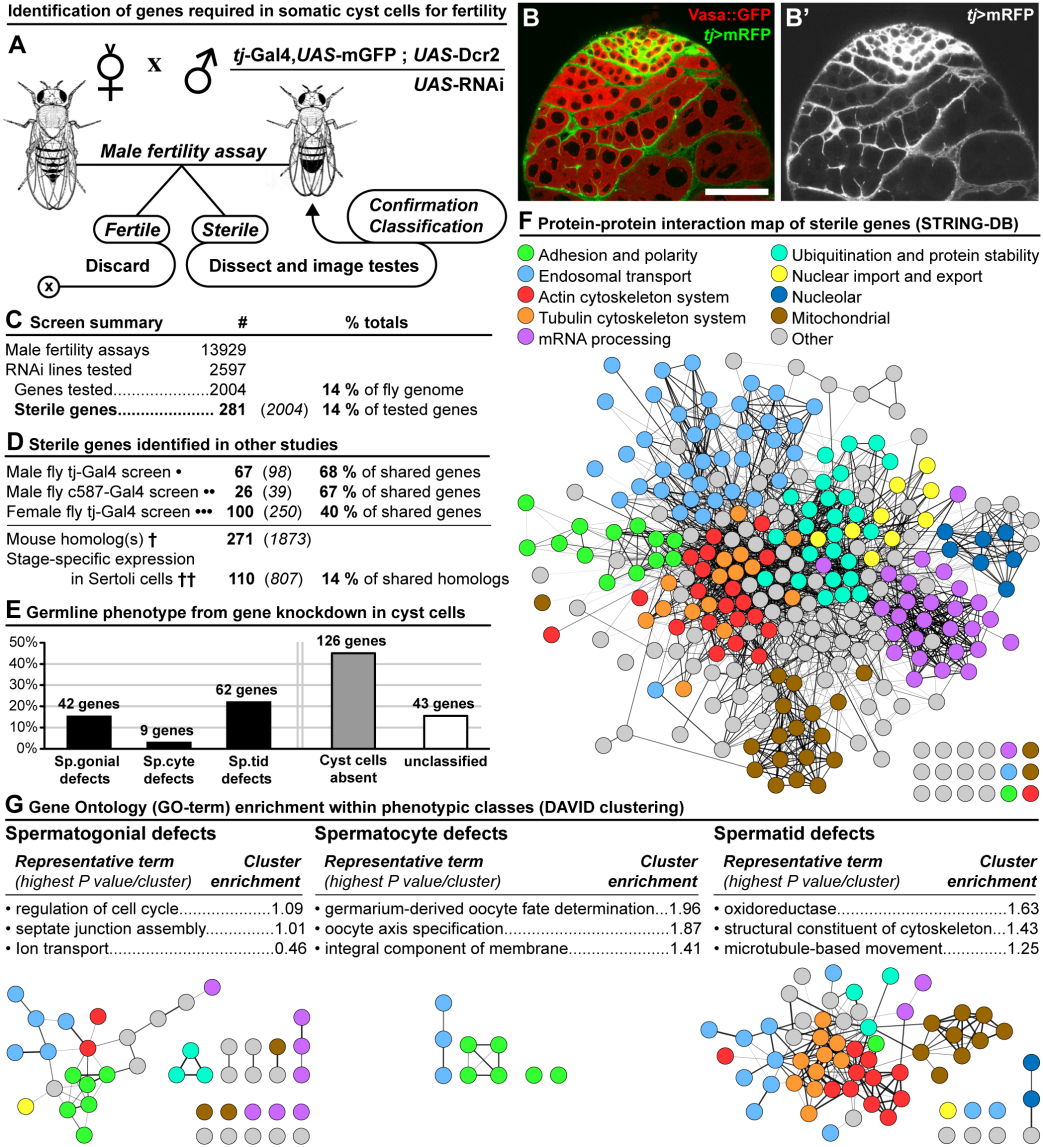
Identification of genes required in cyst cells for male fertility. **(A)** Genetic screen methodology. Fertility assays were used to screen *Drosophila melanogaster* males with RNAi-mediated knockdown of genes in the somatic cyst cells of the testis (*tj*-Gal4 driving expression of UAS-RNAi). **(B)** Expression of the somatic cyst cell marker *tj*-Gal4,UAS-mRFP (*tj*>mRFP) and the germline marker Vasa::GFP in the apical tip of the testis. Scale bar is 50μm. **(C)** General summary of the screen. **(D)** Comparison to related studies in the somatic cells of the gonad. Genes identified in both studies (**bold**) compared to the total possible matching genes (*italics*). Studies included: (**one dot**) *tj*-Gal4>UAS-RNAi in male fly cyst cells [31]; (**two dots**) c587-Gal4>UAS-RNAi in male fly cyst cells [32]; (**three dots**) *tj*-Gal4>UAS-RNAi in female fly follicle cells [33]; (**one dagger**) fly genes with a mouse homolog(s) [34]; (**two daggers**) fly genes with a mouse homolog(s) expressed in a stage specific pattern in Sertoli cells [35]. **(E)** Phenotypic categorization of sterile genes: ‘Sp.gonial (Spermatogonial) defects’ had disruptions of the mitotic germ cell stage; ‘Sp.cyte (Spermatocyte) defects’ had disruptions of the meiotic germ cell stage; ‘Sp.tid (Spermatid) defects’ had disruptions of the morphogenetic germ cell stage; ‘Cyst cells absent’ had no CySCs and no early cyst cells or a complete lack of gonad structures. **(F)** Protein-protein interaction map of sterile genes built using the STRING-Database [36]. Genes have been colour-coded by their function based on a summary of Gene Ontology (GO) terms. **(G)** Most enriched clusters of GO-terms within each phenotypic category determined using the DAVID algorithm [38]. Enrichment calculated for the candidate genes in each phenotypic category compared to the total pool of candidate genes. Sub-networks of the protein-protein interaction map in (F), focusing on each phenotypic category, is shown below the enrichment analysis. See S2 Table for full enrichment analysis. Illustrations in (A) adapted from Thomas Hunt Morgan [74].

To date there has not been a large-scale systematic attempt to identify genes specifically required in the somatic cells of the testis for spermatogenesis. There are two key challenges in such an undertaking: First, the need to study the function of genes exclusively in the somatic cells, and Second, the compounding fact that many genes that mediate spermatogenesis are likely involved in other developmental processes. Consequently, a standard forward genetic screen for mutations causing male sterility would fail to identify many key players in spermatogenesis. With these challenges in mind we designed and optimized a genetic screen using somatic cyst cell specific RNAi-mediated knockdown to identify genes that are required for germline development (Fig 1A and 1B). Spermatogenesis was initially assessed using male fertility assays that were carried out in triplicate. RNAi knockdowns that produced no progeny in the majority of crosses were then retested to confirm the initial result; RNAi knockdowns that consistently showed infertility were analysed phenotypically via immunofluorescence and confocal imaging. The collection of RNAi lines that were screened includes a set of cytoskeletal genes and cytoskeletal regulators, which was initially used as a pilot [26]. Although we enriched the collection for genes associated with stem cell regulation [27–29] the full collection of RNAi lines screened were obtained from a broad array of sources and targeted a wide variety of different gene classes (see S2 Table).

### Summary of screen results

**Fig 2:**
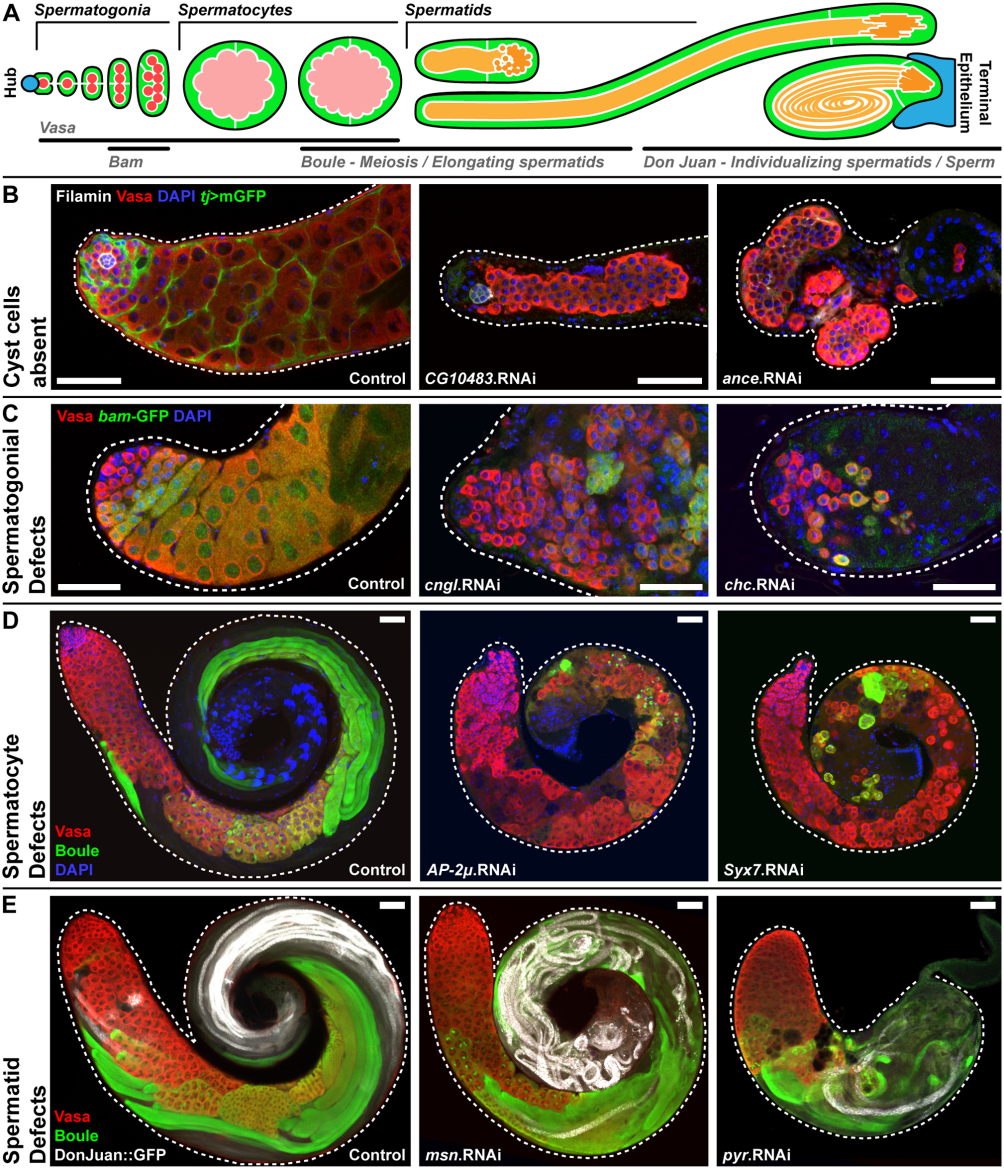
Phenotypic categorization of genes required in cyst cells for male fertility. **(A)** Diagram of soma-germline interactions in the *Drosophila melanogaster* testis illustrating the markers used to characterize germline development at each stage of spermatogenesis. **(B)** Knockdown of genes in the ‘**Cyst cells absent’** category led to loss of cyst cells, while undifferentiated spermatogonia often persisted. Examples include the unnamed gene CG10483 and *angiotensin-converting enzyme* (*ance*). Hub cells labelled by Filamin, cyst cells labelled by *tj*>mGFP, germ cells labelled by Vasa, nuclei labelled by DAPI. **(C)** Knockdown of genes in the ‘**Spermatogonial defect**’ category disrupted mitotic spermatogonial development. Examples include *cyclic nucleotide-gated ion channel-like* (*cngl*) and *clathrin heavy chain* (*chc*). Spermatogonia labelled by Vasa and DAPI, differentiating spermatogonia labelled by *bam*-GFP. **(D)** Knockdown of genes in the ‘**Spermatocyte defec**t’ category disrupted meiotic spermatocyte development. Examples include *adaptor protein complex 2μ* (*AP-2μ*) and *syntaxin 7* (*syx7*). Spermatocytes labelled by Vasa, meiotic spermatocytes labelled by Boule, nuclei labelled with DAPI. **(E)** Knockdown of genes in the ‘**Spermatid defect**’ category disrupted morphogenesis during spermatid development. Examples include *misshapen* (*msn*) and *pyramus* (*pyr*). Early germ cells labelled by Vasa, elongating spermatids labelled by Boule, individualizing spermatids labelled by DonJuan::GFP. Scale bars are 50μm.

Overall we screened 2597 RNAi lines representing 2004 genes, accounting for approximately 14% of the protein-coding genes in the *Drosophila melanogaster* genome [30] (Fig 1C). In total we identified 281 genes (14% of tested genes) as being required in the somatic cyst cells for fertility (see S1 Fig and S1 Table). Each of the 281 candidate genes was knocked down in the cyst cells and the phenotype characterized using markers for the somatic cells (Fig 2; *tj*>mGFP) and the germ cells (Fig 2; Vasa, *bam*-GFP, Boule, DonJuan::GFP). Using the information derived from this analysis each gene knockdown was classified into one of four distinct phenotypic categories (Fig 2B–2E). Although, there was some variability in penetrance of the phenotypes, which is typical for RNAi-mediated knockdown, the classification was based on the most penetrant manifestation of the phenotype. The first category, ‘cyst cells absent’, was characterized by the absence of both CySCs and cyst cells resulting in small rudimentary testes often containing undifferentiated germ cells (Fig 2B). In the second category, ‘spermatogonial defects’, cyst cells were present but the germ cells remained unable to progress past the mitotic stage of development and often over-proliferated as tumour-like growths (Fig 2C). The third phenotypic category, ‘spermatocyte defects’, contained cyst cells as well as germ cells that had begun to differentiate, but were arrested before the completion of the meiotic stage of development (Fig 2D). In the final phenotypic category, ‘spermatid defects’, cyst cells were maintained, but post-meiotic germ cell morphogenesis was disrupted resulting in a failure to produce mature spermatozoa (Fig 2E). Overall, 85% of the candidates were analysed and assigned a phenotypic category. Cyst cells absent represented the largest phenotypic category, accounting for nearly 53% of the classified candidate genes. The proportional breakdown for the other categories is as follows: 18% spermatogonial defects, 4% spermatocyte defects, and 26% spermatid defects (Fig 1E).

### Identification of gene networks required for specific stages of spermatogenesis

To gain more insights from the screen we performed a detailed bioinformatic analysis of the results. First, we compared our screen with similar studies that investigated spermatogenesis in *Drosophila melanogaster* (fly) and *Mus musculus* (mouse) (Fig 1D). For example, two small-scale follow-up screens, subsequent to larger screens, investigated the role of 221 [31] and 113 [32] genes in the somatic cyst cells of the fly testis. Of the shared genes between our screens (genes they identified and we tested), there was approximately a 68% overlap with the candidate genes identified in our screen (67/98 genes and 26/39 genes, respectively) (Fig 1D). Furthermore, we compared our results to a screen recently carried out in the somatic follicle cells of the fly ovary [33]; of the shared genes between our screens there was a 40% overlap in candidate genes (100/250 genes) (Fig 1D). Intriguingly, 271 of our candidate genes have a homologous gene in mice [34] and 110 of these homologs are also expressed in a stage-specific pattern in Sertoli cells during mammalian spermatogenesis [35] (Fig 1D). Together these comparisons confirm that our list of candidates is enriched for genes that regulate somatic cells during spermatogenesis.

To characterize possible molecular interactions and identify genetic networks in our list of candidate genes we built a protein-protein interaction map using the STRING-Database [36] (Fig 1F). Furthermore, we integrated Gene Ontology (GO) terms [37] into our map and found interacting clusters of genes mediating specific cellular functions. This approach identified clusters involved in, adhesion and cell polarity, endosomal transport, the tubulin and actin cytoskeleton systems, mRNA processing, protein ubiquitination and stability, and mitochondrial function (Fig 1F). To determine if any of these gene clusters were associated with specific phenotypes the DAVID algorithm [38] was utilized to identify enriched groups of related GO terms (Fig 1G). This analysis revealed that GO terms associated with septate junction assembly and regulation of the cell cycle were enriched in candidate genes yielding spermatogonial defects. Additionally, GO terms associated with cell polarity and oocyte axis specification were enriched in candidate genes yielding spermatocyte defects, while GO terms associated with the cytoskeleton and microtubule-based movement were enriched in candidate genes yielding spermatid defects (Fig 1G). Overall this demonstrates that the candidate genes identified in our screen contained several interacting gene networks and that these were enriched in specific phenotypic categories.

### Endocytosis modulates niche derived signals to allow cyst cell differentiation

**Fig 3:**
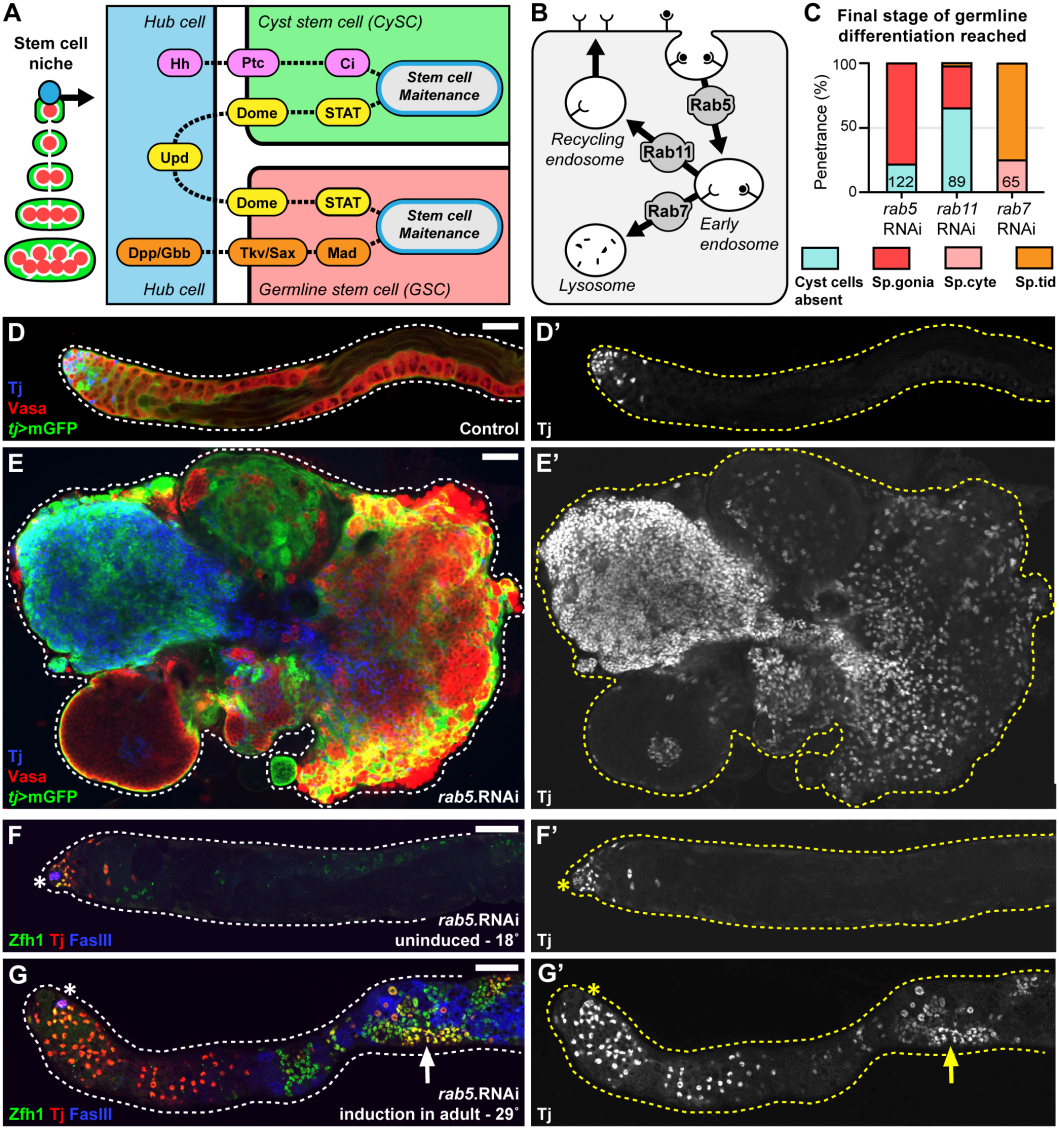
Spermatogonial development requires the endosomal gene Rab5 in cyst cells. **(A)** Diagram of the testis stem cell niche illustrating the signalling pathways used by hub cells to maintain cyst stem cells (CySCs) and germline stem cells (GSCs). The Hedgehog (Hh), Janus Kinase-Signal Transducers and Activators of Transcription (JAK-STAT), and Bone Morphogenetic Protein (BMP) signalling pathways are illustrated using ligands, receptors, and secondary messengers. **(B)** Schematic of receptor mediated endocytosis highlighting the roles of the small GTPases Rab5, Rab7, and Rab11. **(C)** Comparison of phenotypes, represented as final stage of germline differentiation observed, upon cyst cell specific gene knockdown (*tj*>RNAi) of Rab5, Rab7, and Rab11 (number of testes indicated on graph). **(D-E)** Overview of the phenotype resulting from knockdown of Rab5 in somatic cyst cells compared to controls. Both cyst cells and germ cells fail to properly differentiate and develop tumour-like growths. Germ cells labelled by Vasa, cyst cells labelled by membrane-bound GFP (*tj*>mGFP) and nuclear Traffic jam (Tj). **(D’,E’)** Single channel showing Tj. **(F-G)** Adult-specific Rab5 knockdown in cyst cells. Temperature-sensitive Gal80 was used to repress activity of *tj*-Gal4, preventing *rab5*.RNAi expression when raised at 18°C. Hub cells labelled by FasIII, CySCs labelled by Zfh1, and early ‘spermatogonial-stage’ cyst cells labelled by Tj. **(F’,G’)** Single channel showing Tj. **(F)** Adults held at 18°C for 14 days (uninduced). **(G)** Adults shifted to 29°C for 14 days (induced in adult). Scale bars are 50μm.

To elucidate why particular clusters of interacting genes were enriched in specific phenotypic categories representative genes were chosen for further study. The spermatogonial defect category was of special interest since this category likely contained genes that regulate cyst stem cells (CySCs) and germline stem cells (GSCs). Analysis of candidate genes whose knockdown phenotypes manifested during the spermatogonial-stage revealed an intriguing enrichment of genes associated with endocytosis including, Rab5, Chc, Vps16A, Sec15, and AP-1-2β. Since Rab5 has long been established as a master regulator of early endosomes [39] it was chosen for further study as a representative gene within this cluster (Fig 3). Somatic cyst cell specific knockdown of Rab5 resulted in a strong and penetrant phenotype of spermatogenesis arrest at the spermatogonial-stage (Fig 3C). This phenotype was distinct from those observed when other Rab GTPases, such as Rab11 and Rab7 were knocked down, which resulted in earlier and later defects, respectively (Fig 3B and 3C). Rab5 knockdown testes accumulated both somatic cell and germ cell based tumour-like growths over the course of 1 to 2 weeks (Fig 3D and 3E). Based on the observed phenotype we expected the germ cell growths to be arrested at the spermatogonial-stage. To confirm this we examined the morphology of fusomes that interconnect transit-amplifying spermatogonia. This analysis revealed that the germ cell growths associated with Rab5 deficient cyst cells had thin, branched fusomes similar to those found in spermatogonia (S2 Fig). To gain insight into the mechanism responsible for this terminal phenotype, knockdown was induced in adult testes using the conditional temperature-sensitive Gal80 system (Fig 3F and 3G). This approach allowed for the reconstruction of the cellular events preceding the production of the tumour-like growths. Specifically, we observed that when Rab5 was knocked down Zinc-finger homeodomain-1 (Zfh1), a transcription factor essential for CySC identity [40], was expressed in many cyst cells outside the stem cell niche (Fig 3G). By comparison, in control testes Zfh1 is down regulated outside of stem cell niche permitting CySC differentiation (Fig 3F). Sustained Zfh1 expression in cyst cells is linked to accumulation of ectopic CySCs and GSCs [40]. This raises the possibility that the signalling environment around the stem cell niche, which typically mediates the down regulation of Zfh1 to allow CySC differentiation, is disrupted upon Rab5 knockdown.

**Fig 4:**
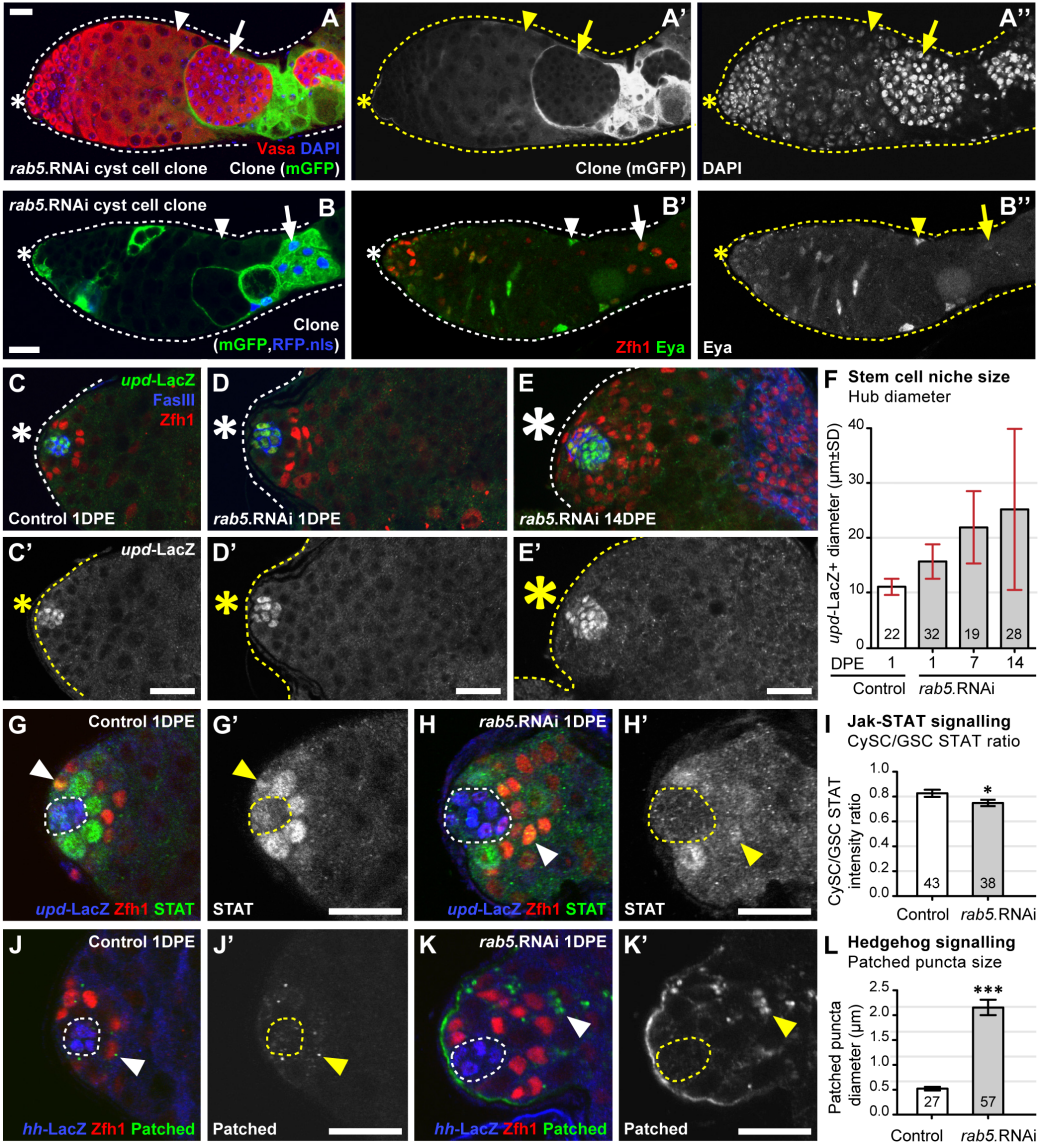
Rab5 regulates cyst stem cell differentiation by modulating niche-derived signalling pathways. **(A)** Cyst cell clones expressing *rab5*.RNAi (labelled by mGFP) encapsulate undifferentiated germ cells (labelled by Vasa and DAPI; **Arrow**). **(B)** Cyst cell clones expressing *rab5*.RNAi (labelled by mGFP and RFP.nls; **Arrow**) maintain expression of the CySC marker Zfh1 and do not express the differentiated cyst cell marker Eya. **(C-E)** Changes in the size of the stem cell niche upon knockdown of Rab5 in cyst cells. Representative images of the stem cell niche with hub cells labelled by both *upd*-LacZ and FasIII, and CySCs labelled by Zfh1. **(F)** Quantitation of hub sizes between 1 and 14 days post eclosion (DPE) measured as the maximum diameter of *upd*-LacZ positive cells. (Number of testes indicated on graph; mean±SD). **(G-K)** Changes in JAK-STAT and Hh signalling after RNAi-mediated Rab5 knockdown in cyst cells. **(G-H)** Hub cells labelled using the JAK-STAT ligand reporter *upd*-LacZ, CySCs labelled by Zfh1, JAK-STAT signalling detected by STAT accumulation in CySCs (**Arrowheads**). **(I)** Quantitation of JAK-STAT signalling measured as the fluorescent intensity of JAK-STAT in CySC nuclei compared to the intensity in GSCs. (Number of CySCs indicated on graph; mean±SEM; student t-test p-value<0.05). (**J-K**) Hub cells labelled by the Hedgehog ligand reporter *hh*-LacZ, CySCs labelled by Zfh1, Hedgehog signalling marked by Patched accumulation in CySCs (**Arrowheads**). **(L)** Quantitation of Hedgehog signalling measured as the maximum diameter of Patched puncta. (Number of testes indicated on graph; mean±SEM; student t-test p-value<0.001). Scale bars are 20μm.

We envisaged two possible mechanisms by which Rab5 knockdown could disrupt the signalling environment around the niche such that cyst cell differentiation is blocked. The niche itself could be altered, thus blocking differentiation, or Rab5 knockdown could inhibit the ability of cyst cells to processes niche-derived signals. Two pieces of evidence argue the latter. First, the effect of Rab5 was cell autonomous as shown by clonal RNAi-mediated knockdown of Rab5 (Fig 4A and 4B). In spermatocysts containing somatic clones expressing *rab5*.RNAi the cyst cells encapsulated the germ cells but both tissues failed to fully differentiate (Fig 4A). This phenotype was less penetrant then that observed when Rab5 was knocked down in all cyst cells, which may reflect differences in the level of knockdown due to the use of an alternative Gal4 (c587-Gal4 in clones versus *tj*-Gal4 in the constitutive knockdown). Alternatively this could reflect differences due to the late induction of the knockdown specifically in adult clones. Nonetheless, the stem cell marker Zfh1 was often expressed in these somatic cell clones while the differentiation marker Eyes absent (Eya) [41] was not expressed (Fig 4B). Second, although the stem cell niche underwent abnormal growth in Rab5 knockdown flies, in testes from 1-day post eclosion (DPE) males the hub was only marginally larger than controls (Fig 4C and 4F). Taken together these results suggest that Rab5 modulates the ability of CySCs to process stem cell renewal signals and enables cyst cells to differentiate as they leave the niche.

Multiple niche-derived signals have been implicated in maintenance of cyst stem cell (CySC) fate and sustained Zfh1 expression (Fig 3A). In particular, it is known that JAK-STAT signalling maintains Zfh1 expression in CySCs [40]. Based on this, one prediction to explain the Rab5 knockdown phenotype is that JAK-STAT signalling levels are higher in the CySCs. Surprisingly this is not the case as measurement of STAT immunostaining revealed levels that were slightly lower than controls (Fig 4G–4I). However ectopic JAK-STAT signalling was detected in the tumour-like growths of cyst cells found in aged males, suggesting abnormal activation of this pathway could be driving Zfh1 expression outside the niche (S3E Fig). The Hedgehog (Hh) pathway also plays a role in maintaining Zfh1 expression and promoting CySC identity [42,43]. To measure Hh signalling we analysed the localization of the Hh receptor Patched as it has been shown to be a useful readout for Hh signalling in CySCs [44]. Loss of Rab5 induces a substantial increase in Hh signalling as judged by the size of Patched staining puncta (Fig 4J–4L). Furthermore Hh signalling was also detected in the tumour-like growths of cyst cells that form outside of the niche in aged males (S3D Fig). Overall this data suggests that Rab5 modulates signalling pathways, such as JAK-STAT and Hh, to enable cyst cell differentiation.

Both JAK-STAT signalling and Hh signalling have been implicated in controlling the expression of BMP ligands by CySCs [44,45]. Ectopic expression of the BMP ligand Decapentaplegic (Dpp) also leads to the over-proliferation of spermatogonia [46]. We therefore examined whether BMP signalling was elevated in the germ cells associated with Rab5 deficient cyst cells by immunostaining for the phosphorylated form of Mothers against decapentaplegic (pMad). While pMad is only weakly detected in controls it is robustly detected in spermatogonia when Rab5 is knocked down in the cyst cells (S3A–S3C Fig). Together with our previous results this demonstrates that Rab5 mediates the differentiation of cyst cells and regulates their expression of BMP ligands to facilitate spermatogonial development.

### Cell polarity regulates the adluminal domain of the spermatocyst

**Fig 5:**
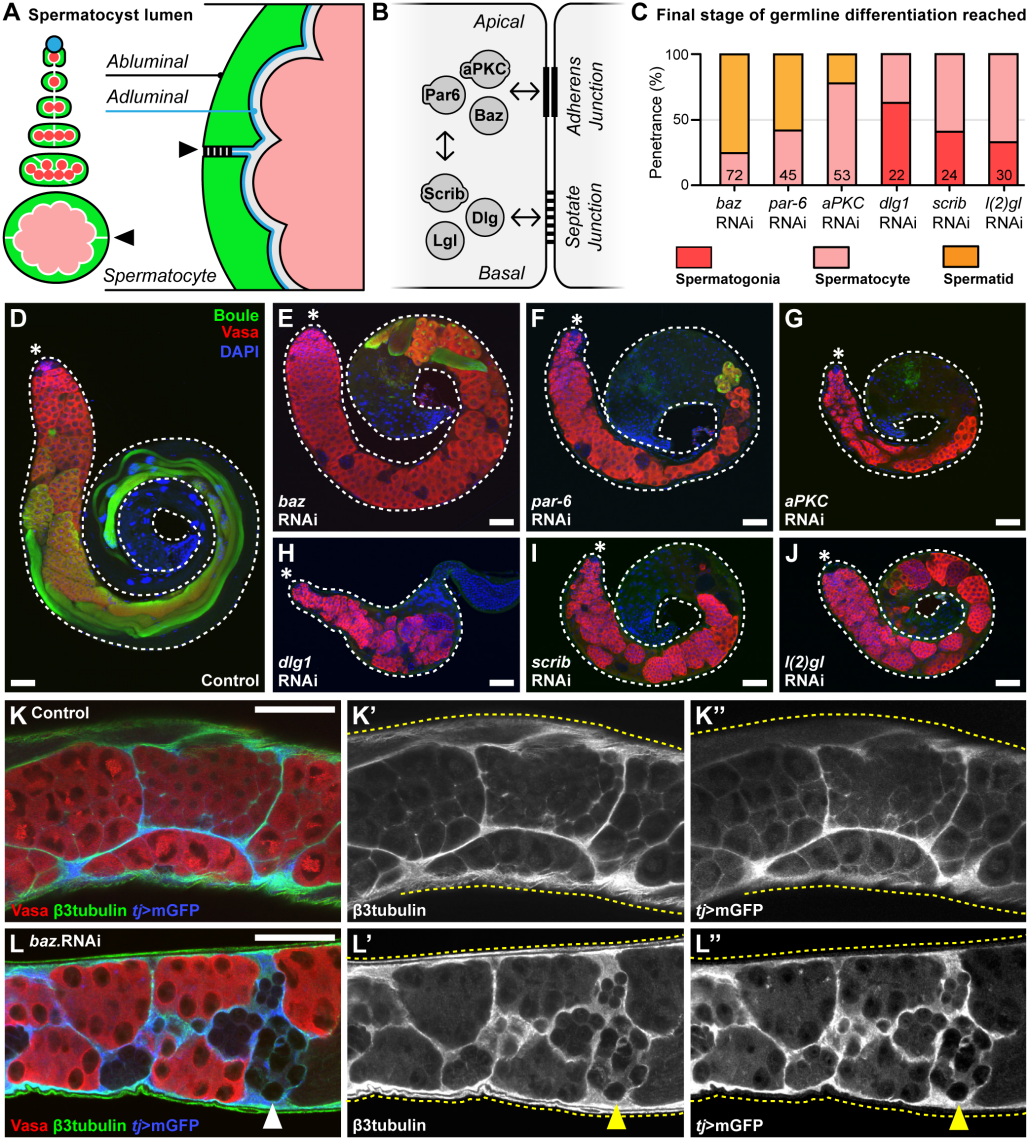
Spermatocyte development requires the polarity genes Baz, Par-6, and aPKC in cyst cells. **(A)** Diagram illustrating the topology of the spermatocyst, with two cyst cells (green) encapsulating the germ cells (pink) forming an inner adluminal surface (germline-facing) and outer abluminal surface (environment-facing). **(B)** Schematic of the interactions between cell-cell adhesions, the Par cell polarity module (Baz, Par-3, aPKC), and the Scribble cell polarity module (Dlg1, Scrib, L(2)gl). **(C)** Final stage of germline differentiation observed with cyst cell specific gene knockdown (*tj*>RNAi) of different polarity module genes (number of testes indicated on graph). **(D-J)** Overview of the phenotype resulting from knockdown of Par and Scribble module genes in somatic cyst cells compared to controls. Early germ cells labelled by Vasa, meiotic germ cells labelled by Boule, and nuclei labelled by DAPI. **(E-G)** Knockdown of Par module genes in cyst cells yields spermatocyte-stage defects. (**H-J**) Knockdown of Scribble module genes in cyst cells yields spermatogonial-stage defects. **(K-L)** Cyst cells (labelled by β3tubulin and *tj*>mGFP) encapsulate dying spermatocyte-stage germ cells (identified by loss of Vasa staining; **Arrowhead**) when the Par polarity module gene Baz is disrupted. **(K’,L’)** Single channel showing β3tubulin. **(K”,L”)** Single channel showing *tj*>mGFP. Scale bars are 50μm.

Of all the phenotypic categories defined in the screen, the spermatocyte defects category was the smallest, consisting exclusively of regulators of cell polarity and polarized protein trafficking. The phenotypes resulting from cyst cell specific knockdown of candidate genes in this functional cluster were analysed in detail to understand their role during the spermatocyte-stage. Specifically, our analysis focused on the role of a group of interacting proteins composed of Bazooka (Baz), Par-6, and atypical Protein Kinase C (aPKC). Together these proteins function as the ‘Par polarity module’ and has long been established as a central regulator of apical polarity in epithelial cells [47] (Fig 5B). Moreover, Par module genes are expressed in cyst cells during spermatogenesis [48,49]. When the Par module genes (Baz, Par-6, aPKC) were knocked down in cyst cells the spermatogonial-stage appeared normal and the germ cells were encapsulated similar to controls (Fig 5E and 5F, 5K–5L). However, although the germ cells often entered the meiotic spermatocyte-stage, as judged by the expression of Boule [50], spermatogenesis arrested shortly afterwards (Fig 5E and 5F). This phenotype does not represent a general consequence of disrupting cell polarity. For example, in agreement with previous results [51], we found that knockdown of the ‘Scribble polarity module’ genes (Dlg1, Scrib, L(2)gl) that regulate baso-lateral polarity in epithelial cells [52] gave rise to a distinct phenotype that manifested during the mitotic spermatogonial-stage (Fig 5H–5J). In contrast, the main phenotype observed upon cyst cell specific knockdown of Par polarity module genes (Baz, Par-6, aPKC) was a failure of the germ cells to develop or survive past the meiotic spermatocyte-stage (Fig 5E and 5F, 5K–5L).

**Fig 6:**
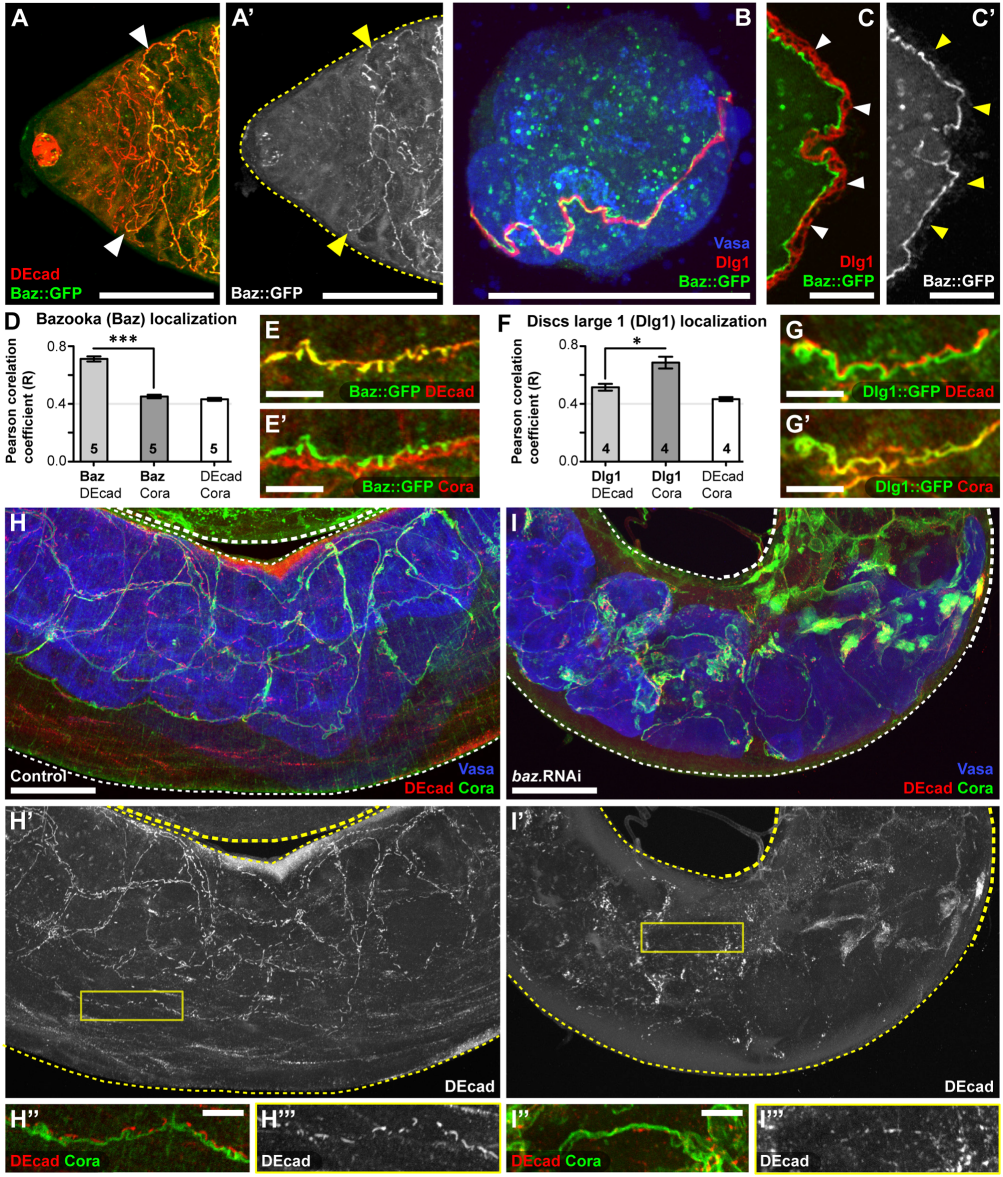
Bazooka maintains polarized adherens junctions between the encapsulating cyst cells of each spermatocyst. **(A)** The Par polarity protein Baz (labelled by Baz::GFP) prominently localizes to adherens junctions (labelled by DEcad) during the spermatocyte-stage of spermatogenesis (**Arrowheads**). **(B-C)** Localization of Baz (Baz::GFP) and the Scribble module protein Dlg1 in individual spermatocysts isolated from the testis.**(B)** During the early spermatocyte-stage both Dlg1 and Baz::GFP are localized to a junctional belt connecting the two cyst cells that encapsulate the germline (labelled by Vasa). **(C)** By the late spermatocyte-stage Dlg1 and Baz::GFP are segregated into distinct polarized domains. The inner adluminal surface (germline-facing) is to the left and the outer abluminal surface (environment-facing) is to the right (**Arrowheads**). **(D-E)** Quantification and representative images of the junctional belt between cyst cells show that the Par polarity protein Baz (Baz::GFP) predominantly co-localizes with DE-cadherin (DEcad), a marker of adherens junctions. (Number of testes indicated on graph; mean±SEM; student t-test p-value<0.001). **(F-G)** Quantification and representative images of the junctional belt between cyst cells show that the Scribble polarity protein Dlg1 (Dlg1::GFP) predominantly co-localizes with Coracle (Cora), a marker of septate junctions. (Number of testes indicated on graph; mean±SEM; student t-test p-value<0.05). **(H-I)** Knockdown of Baz leads to disruption of DE-cadherin (DEcad) based junctions. Spermatocyte-stage spermatocysts in the mid-region of the testes with germ cells labelled by Vasa, septate junctions labelled by Cora, and adherens junctions labelled by DEcad. **(H”,I”)** Close-up images of the junctional belt between cyst cells in a single spermatocyst within the testes (Boxes in H’,I’). All images are *z*-projections. Scale bars are 50μm (A-B,H-H’,I-I’) and 10μm (C,E,G,H”-H”‘,I”-I”‘).

To elucidate the specific cellular role of Par polarity module genes during the spermatocyte-stage, the subcellular distribution of Bazooka (Baz) was examined (Fig 6A–6C). Baz expression becomes prominent by the early spermatocyte-stage and is localized to the junctional belt between encapsulating cyst cells [53] (Fig 6A and 6B and S4 Fig). Furthermore, Baz was observed to occupy a distinct junctional complex compared to the Scribble module protein Discs large-1 (Dlg1) (Fig 6C). This shows that during the spermatocyte-stage, cyst cells exhibit a form of cell polarity that utilizes two spatially separated regulatory modules. A key role of the Par polarity module in epithelial cells is to coordinate the establishment and maintenance of apico-lateral adherens junctions [47]. In comparison, the Scribble polarity module is required to maintain baso-lateral septate junctions [52]. These roles were recapitulated in the cyst cells, even though these cells are not epithelia (Fig 6D–6G). Specifically, the Par module protein Bazooka (Baz::GFP) predominantly colocalizes with the adherens junction component DE-Cadherin (DEcad) (Fig 6D and 6E), while the Scribble module protein Discs large-1 (Dlg1::GFP) predominantly colocalizes with the septate junction component Coracle (Cora) (Fig 6F and 6G). Importantly, knockdown of Baz resulted in the disruption of adherens junctions (DEcad), but not septate junctions (Cora) in spermatocyte-stage cyst cells (Fig 6H and 6I). These results suggest that mechanisms regulating cell polarity are essential for spermatocyte development and survival within the adluminal compartment of the spermatocyst.

### Microtubule-based transport is required for cyst cell morphogenesis

**Fig 7:**
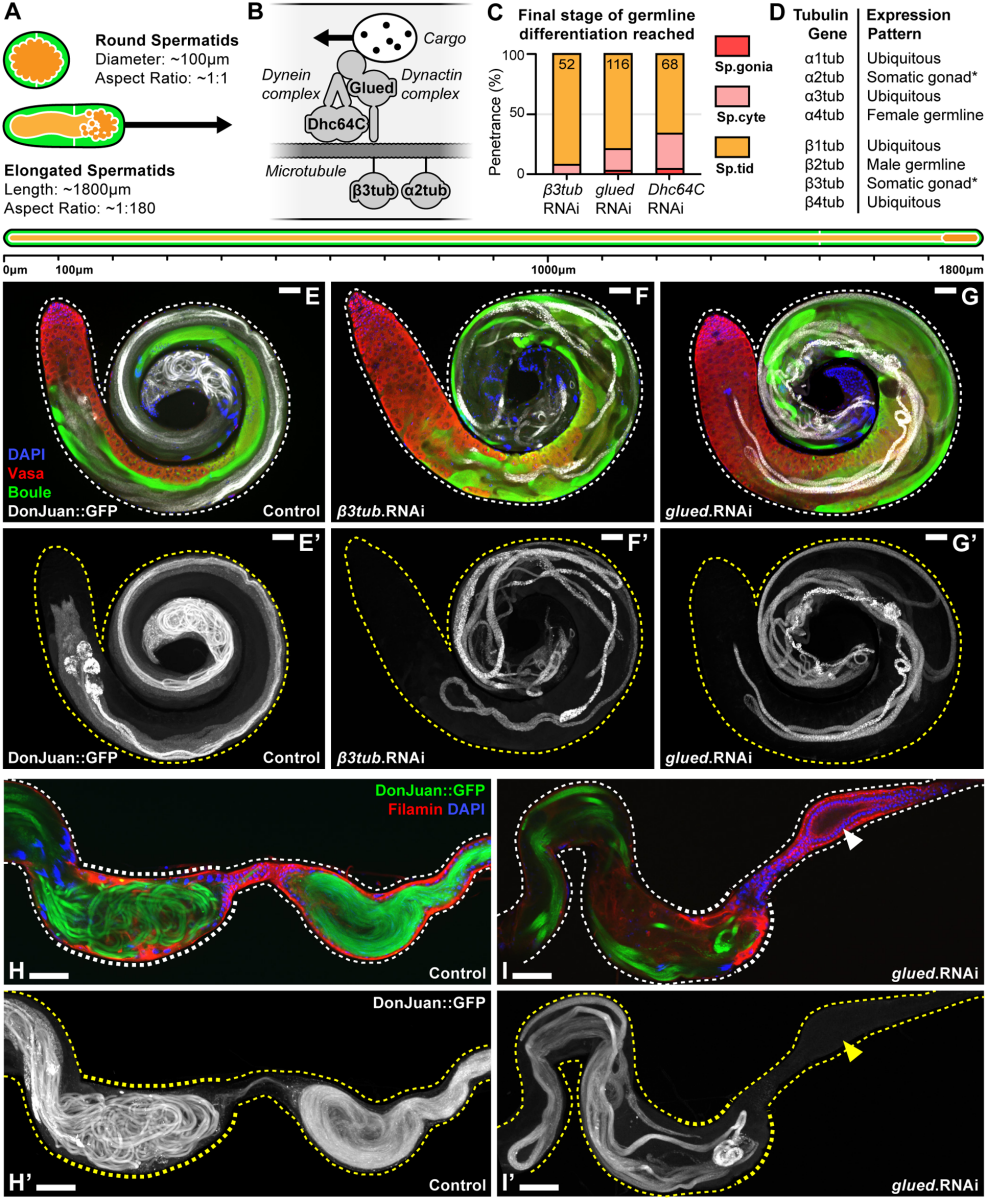
Spermatid development requires β3tubulin and the dynein-dynactin complex in cyst cells. **(A)** Diagram of the spermatocyst at the round spermatid-stage through to the fully elongated spermatid-stage. **(B)** Schematic illustrating dynein (Dhc64C) mediated transport of dynactin (Glued) tethered cargo along microtubules (α2tubulin and β3tubulin). **(C)** Comparison of phenotypes, represented as final stage of germline differentiation observed, upon RNAi-mediated cyst cell specific gene knockdown (*tj*>RNAi) of microtubule subunits (β3tubulin), or the dynein (Dhc64C) and dynactin (Glued) complex (number of testes indicated on graph). **(D)** Adult expression pattern of the α and β tubulins. (**Asterisks**) In adult males both α2tubulin and β3tubulin are expressed exclusively in the somatic cells of the testis [55,56]. **(E-G)** Representative phenotypes resulting from cyst cell specific knockdown of β3tubulin, or the dynactin complex component Glued. The early germ cells are labelled by Vasa, elongating spermatids labelled by Boule, individualizing spermatids labelled by DonJuan::GFP, and nuclei labelled by DAPI. **(H-I)** Knockdown of Glued in cyst cells results in the failure of individualized spermatozoa to reach the seminal vesicle (**Arrowhead**). The terminal epithelium and seminal vesicle are both labelled by Filamin, individualizing spermatids labelled by DonJuan::GFP, and nuclei labelled by DAPI. Full *z*-projections of DonJuan::GFP (E’,F’,G’,H’,I’). Scale bars are 50μm.

**Fig 8:**
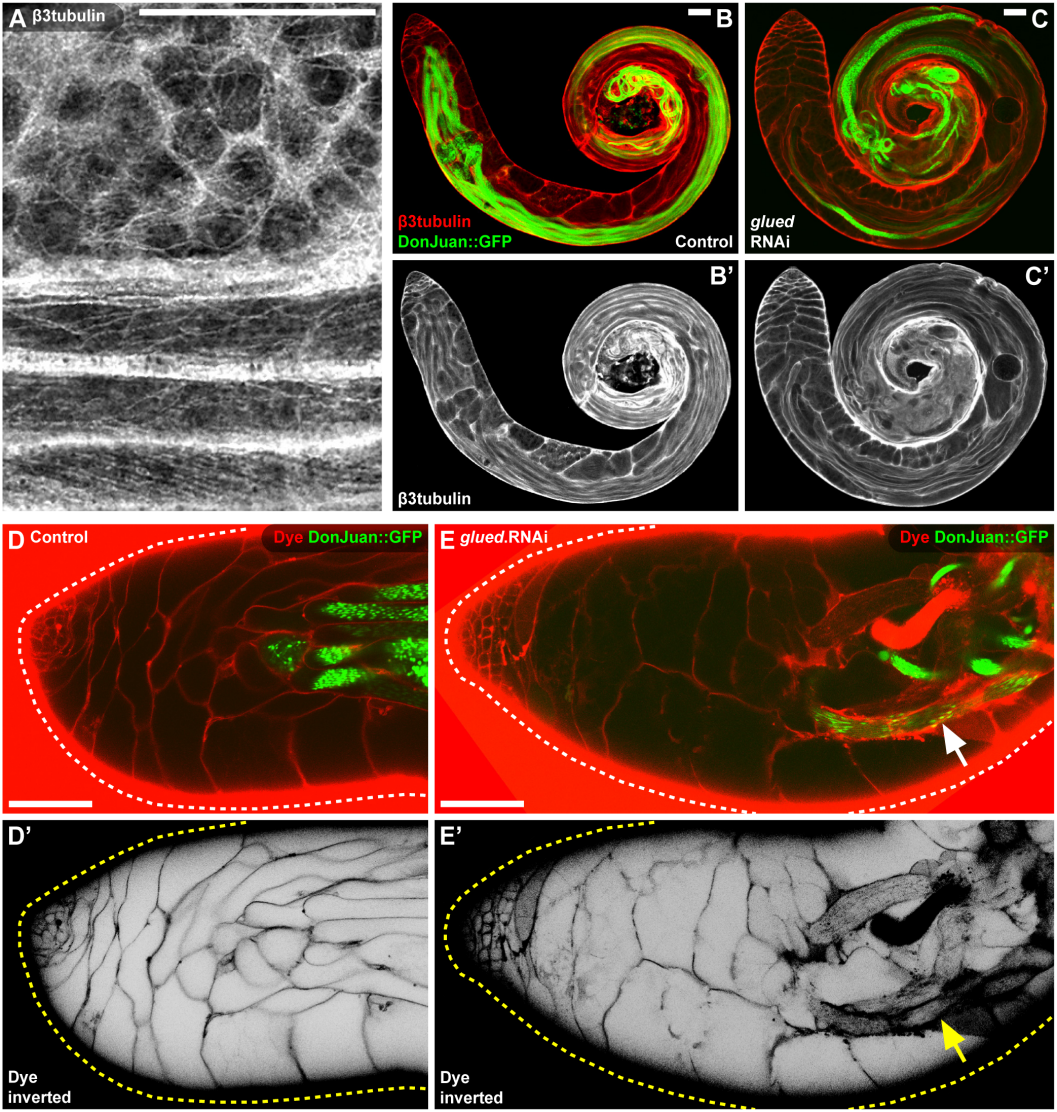
Microtubule-based transport is required to maintain the structural integrity of the spermatocyst. **(A)** Rearrangement of the cyst cell microtubule cytoskeleton (labelled by β3tubulin) between the round spermatid-stage (Top) and the elongated spermatid-stage (Bottom). **(B-C)** Knockdown of Glued in cyst cells does not disrupt the encapsulation of spermatogonia or spermatocytes but does disrupt the microtubule cytoskeleton during the spermatid-stage. Cyst cells labelled by β3tubulin and elongated spermatids labelled by DonJuan::GFP. **(B’,C’)** Single channel showing β3tubulin. **(D-E)** The integrity of the spermatid-stage spermatocysts is compromised by knockdown of Glued in the cyst cells. Permeability assay of spermatid-stage spermatocysts reveals that fluorescent dye added to the environment (Dye) is able to access the surface of elongated spermatids (labelled by DonJuan::GFP) when Glued is knocked down. **(D’,E’)** Single channel showing fluorescent dye with the intensity inverted. Scale bars are 50μm.

The final phenotypic category defined by the screen, the spermatid defects category, contained a striking enrichment of candidate genes associated with microtubule-based transport. Amongst the genes in this category were components of the dynein (Dhc64C) and dynactin (Glued) complex [54]. This category also contained the β3 (β-tubulin at 60D) and α2 (α-tubulin at 85E) microtubule subunits, both of which are expressed exclusively in the somatic cells of the testis in adult males [55,56] (Fig 7B and 7D). Knockdown of these genes in cyst cells resulted in fewer fully elongated spermatids, as judged by the expression of Don Juan [57], even after normal progression through the spermatogonial and spermatocyte-stages (Fig 7C, 7E and 7F). As a result of these defects, individualized spermatozoa were not extruded into the seminal vesicle (Fig 7H and 7I). In the germline, it is well established that microtubules and the dynein-dynactin complex are central to spermatid elongation [58,59]. To gain insight into the role of these proteins in cyst cells, the expression of β3tubulin was characterized during the spermatid-stage (Fig 8A). This revealed a striking rearrangement of the microtubules from the short arrays typical of ‘round spermatid-stage’ cyst cells to the extended, linear arrangement typical of ‘elongated spermatid-stage’ cyst cells (Fig 8A). While cyst cell specific knockdown of the dynactin component Glued did not disrupt the initial encapsulation of the germline, it did disrupt the dense arrangement of β3tubulin in spermatid-stage cyst cells (Fig 8B and 8C). These results were consistent with a role for microtubules and the dynein-dynactin complex in cyst cell morphogenesis during spermatid development. To understand the underlying phenotype resulting from knockdown of Glued in the cyst cells, spermatocyst integrity was analysed using a permeability assay. This assay measures the ability of fluorescently-conjugated dextran to access the surface of germ cells at different stages of development [53]. In control testes, the dye was unable to access the surface of spermatid-stage germ cells in the apical tip of the testis, consistent with an intact spermatocyst (Fig 8D). By comparison, when Glued was knocked down in cyst cells, the dye was able to access the surface of fully elongated spermatids that had reached the apical tip of the testis (Fig 8E). Overall, this data suggests that microtubules and the dynein-dynactin complex play a key role in maintaining the growth and integrity of cyst cells during spermatid development.

## Discussion

We performed an RNAi-mediated knockdown screen and identified candidate genes that are required in the somatic cyst cells of the *Drosophila melanogaster* testis for spermatogenesis. Analysis of the candidates revealed functional gene networks that were required for specific stages of spermatogenesis. Phenotypic analysis of these genes provides insight into the mechanisms that are essential for somatic cells to support each stage of germline development. In particular, we show that components of the endocytic machinery are critical for spermatogonial development. Endocytosis modulates the activity of niche-derived signals and is thus essential for regulating cyst stem cell differentiation. Furthermore, proteins that maintain cell polarity and regulate cell-cell junctions in epithelia serve a similar role in the cyst cells during spermatocyte development. Failure to maintain cell polarity leads to the disruption of adherens junctions and results in the death of spermatocytes within the adluminal domain of the spermatocyst. Finally, we demonstrate that microtubule-based transport is required in cyst cells for the completion of spermatid development. A specific set of microtubule subunits and the dynein-dynactin complex mediate the large morphological changes in cyst cells during spermatid elongation. Together these results provide mechanistic insight into the cell biological events that underlie spermatogenesis and identify functional clusters of genes that act in the somatic cells to mediate germline development.

Spermatogenesis involves a sequence of coordinated morphogenetic behaviours. Both germ cells and somatic cells undergo enormous changes in cell shape and size, but the molecular mechanisms underlying these events, particular those of the somatic cells, are not well understood. Our screen now provides entry points in attempting to understand these molecular mechanisms. It will be possible, starting with the candidate genes we identified, to mine protein interaction databases and identify other candidate genes that act during each stage of spermatogenesis. Importantly, although we have assigned each of our candidates a phenotypic category based on the stage at which spermatogenesis arrested, it is likely that many of these genes act during multiple stages of spermatogenesis. While our study assigns a function to these genes at a particular stage, this might only reflect the time at which RNAi mediated gene knockdown becomes effective. Also, it is important to note that the phenotypes we observe might reflect phenotypes that arise during earlier stages or in embryonic development, but only manifest later on. For these reasons, follow up experiments on these candidate genes will need to use, as we have done (see Figs 3G and 4B), clonal and inducible knockdown approaches [60] to confirm the stage at which they are required. Nonetheless, for the genes we identified in our screen that have known functions in spermatogenesis there was a good correspondence between their previously studied roles and the stage at which our analysis suggested they were required.

Our results suggesting that Rab5 functions in the somatic cyst cells to fine-tune niche-derived stem cell maintenance signals are supported by recent analysis of the role Rab5 plays in modulating expression of the BMP ligand Decapentaplegic (Dpp) [61]. While we show that Rab5 controls the levels of JAK-STAT and Hh signalling in cyst cells, Rab5 has similarly been shown to control the levels of JAK-STAT and Jun kinase (JNK) signalling as well as Dpp expression in cyst cells [61]. Rab5 and the endosomal machinery are likely to be key regulatory nodes for controlling how cyst cells receive, process, and transmit signals with the stem cell niche and the germline. For both Hh and JNK signalling it appears the predominant role of Rab5-mediated endocytosis is to attenuate signalling activity in the cyst cells. While the role Rab5 plays in regulating JAK-STAT signalling appears to be more complex. Perturbation of the Rab5 endocytic machinery modulates cyst cells sensitivity or responsiveness to niche-derived signals and maintains them in an undifferentiated state for a longer period of time. As a consequence of this, the cyst cells produce tumour-like growths exhibiting characteristics of stem cell identity, such as Zfh1 and Traffic Jam (Tj) expression [40,62]. Overall, these results demonstrate how cell-cell signalling regulates the transition from stem cell to differentiating cell, which represents a hallmark of the spermatogonial-stage. Indeed, another gene cluster enriched in this phenotypic category, septate junction components, has previously been shown to modulate soma-germline signalling in the testis [53,63]. We expect that analysis of other gene clusters enriched in this phenotypic category is likely to yield more insight into the mechanisms that mediate cyst cell differentiation.

Mechanisms that regulate cell polarity and adhesion are known to play a role in spermatogenesis; in particular they have important functions in the germline. During early spermatogenesis the Par module protein Baz associates with centrosomes in germline stem cells and this interaction is key for ensuring proper asymmetric cell division [64]. Another Par module protein, aPKC, has been shown to be essential for germline polarization during the spermatid-stage of development [49]. Although germ cells are not epithelial, they do exhibit a form of polarization with spermatid nuclei localized to one side of the spermatocyst surrounded by the head cyst cell, while the flagellar axonemes grow towards the other side surrounded by the tail cyst cell [11]. We find that in somatic cyst cells, polarization mechanisms are required even earlier during the spermatocyte-stage. Our work builds on these previous studies and adds three insights. First, we provide evidence that the polarity cyst cells exhibit is reminiscent of epithelial polarization, with multiple, spatially segregated polarity modules. Second, we find that in spermatocyte-stage cyst cells the Par polarity module is specifically required for the maintenance of adherens junctions. Third, we find that different polarity modules have different roles, with the Scribble module regulating spermatogonial development and the Par module regulating spermatocyte development. The Par module may mediate the substantial growth in volume of the spermatocyst lumen as this would likely require cell-cell junctional remodeling [65,66]. Together these findings suggest that the gene clusters enriched in this phenotypic category mediate the formation or maintenance of the cyst cell luminal environment.

Microtubules and the dynein-dynactin complex are known to play important roles in the germline during spermatogenesis. Mutations in the germ cell specific β2tubulin subunit [58] or components of the dynein–dynactin complex [59,67–69] affect the formation of the flagellar axonemes. It is also known that mutations in the somatic cell specific β3tubulin subunit results in male sterility [70]. We show that in cyst cells both β3tubulin-based microtubules and the dynein-dynactin complex are required to maintain the integrity of the spermatocyst during spermatid development. One possibility is that as spermatid elongation is independent of the soma [71] the cyst cells must grow and change shape simply to accommodate the dramatic increase in the length of the germ cells. Specific microtubule subtypes and the dynein-dynactin complex may act as the morphogenetic machinery that drives the elongation of cyst cells and in their absence the structure of the spermatocyst is compromised. Our findings are consistent with the interpretation that many of the gene clusters enriched in this phenotypic category are important regulators of cyst cell morphogenesis.

Our work adds to a growing list of studies that explore spermatogenesis using the powerful genetic and imaging tools available in *Drosophila melanogaster*. Two recent studies used similar RNAi-based approach to ours, but focused predominantly on genes required in the germline [31,32]. By contrast our study is the largest to focus specifically on how the somatic cells of the testis regulate spermatogenesis. Our work thus provides a comprehensive overview of the complex cooperation between the soma and the germline required for the remarkable process of spermatogenesis.

## Materials and methods

### Screen

Female flies were collected from *tj*-Gal4,UAS-mGFP;UAS-Dcr2 or *tj*-Gal4;UAS-mRFP,UAS-Dcr2 stocks and bred to a library of males from UAS-RNAi stocks. Individual male progeny (*tj*>RNAi) were transferred to vials with three to five virgin w^1118^ females for fertility assays. Virgin females were collected from the Virginator stock [♀*w*^1118^/ *w*^1118^ and ♂*w*^1118^/Dp(2;Y)G,*hs*-hid], raised at 20°C and shifted to 29°C for 3 days during pupation to kill males. Individual male fertility assays were done in triplicate and listed as sterile if no larva/pupae were present 14 days post-mating. If the majority of fertility assays yielded sterile males, more males were tested and additional RNAi lines targeting the gene were screened.

### Phenotypic classification

Spermatogonia were identified by expression of Vasa, dense DAPI staining nuclei, and their small size; differentiating spermatogonia were identified by expression of both Vasa and *bam*-GFP. Spermatocytes were identified by expression of Vasa, dispersed DAPI staining, and their large size; meiotic spermatocytes identified by expression of both Vasa and Boule. Early spermatids identified by expression of Boule, polarization of their nuclei, and their elongated shape; individualizing spermatids identified by expression of DonJuan::GFP. Spermatozoa identified by expression of DonJuan::GFP and their accumulation in the seminal vesicle as individual cells. Phenotypes were assigned based on the stage of germline development that was disrupted in the majority of samples. Germline disruption was defined as over-proliferation, disintegration, death, or other disorders the germ cells including the failure of spermatozoa to reach the seminal vesicle.

### Bioinformatics

Protein-protein interaction network (Fig 1F, 1G and S1 Fig) built using STRING-DB 10.0 [36]. Network map created using Cytoscape 3.0 [72] and the force-directed layout mediated by the ‘AllegroLayout’ plugin. Sterile genes were coloured by their functional class based on a summary of the associated GO terms and the relevant literature by the authors. GO term enrichment (Fig 1G) was assessed using DAVID 6.8 [38] by comparing sterile genes with either spermatogonial, spermatocyte, or spermatid defects to all the sterile genes identified. DAVID analysis included the ‘GO DIRECT’ terms for Biological Process (BP), Cell Compartment (CC), and Molecular Function (MF), as well as ‘UP KEYWORDS’ and ‘UP SEQ FEATURE’ (Fig 1G and S2 Table).

### Genetics

*tj*-Gal4 (P-element insertion {GawB}NP1624) obtained from the Bloomington Drosophila Stock Center [BDRC]). Additional lines from the BDSC included the Virginator (*w*^1118^/Dp(2;Y)G,*hs*-hid), UAS-Dcr2 (UAS-Dicer2), UAS-mGFP (UAS-mCD8::GFP), UAS-mRFP (UAS-mCD8::Tomato), UAS-RFP.nls (UAS-RedStinger), Baz::GFP (*baz*^CC01941^), Dlg1::GFP (*dlg1*^YC0005^), DonJuan::GFP (*dj*-GFP.S), and *hh*-LacZ (*hh*^P30^). Vasa::GFP (*vas*.EGFP.HA) provided by the Kyoto Drosophila Genetic Resource Center (DGRC). *upd*-LacZ (*upd1*-PD) courtesy of David Bilder, University of California Berkeley, USA. *bam*-GFP (*bam*-GFP^-799/+133^) courtesy of Christian Bökel, Center for Regenerative Therapies Dresden, Germany. UAS-RNAi lines obtained from the Transgenic RNAi Project (TRiP), the Vienna Drosophila RNAi Centre (VDRC), and the National Institute of Genetics (NIG). RNAi lines used in Figs 2–8 include UAS-*CG10483*.RNAi (TRiP.HMS01023), UAS-*ance*.RNAi (TRiP.HMS03009), UAS-*AP-2μ*.RNAi (TRiP.JF02875), UAS-*syx7*.RNAi (TRiP.JF02436), UAS-*msn*.RNAi (TRiP.HMJ02084), UAS-*pyr*.RNAi (VDRC.36523), UAS-*rab5*.RNAi (VDRC.103945 except Frt40A;VDRC.34096 in Fig 4A and 4B), UAS-*rab11*.RNAi (TRiP.JF02812), UAS-*rab7*.RNAi (VDRC.40338), UAS-*baz*.RNAi (VDRC.2914), UAS-*par-6*.RNAi (VDRC.108560), UAS-*aPKC*.RNAi (VDRC.2907), UAS-*dlg1*.RNAi (TRiP.JF01365), UAS-*scrib*.RNAi (TRiP.JF03229), UAS-*l(2)gl*.RNAi (TRiP.HMS01522), UAS-*β3tub*.RNAi (VDRC.34607), UAS-*glued*.RNAi (TRiP.JF02803), UAS-*Dhc64C*.RNAi (TRiP.JF03177). See S2 Table for all RNAi stocks.

### Immunohistochemistry

Samples fixed using 4% PFA in PBS for 15 minutes. Antibodies incubated in PBS supplemented with 0.2% BSA and 0.3% Triton-X at 4° with the exception of rabbit-anti-STAT incubated at 20°. Primary antibodies included rat-anti-Filamin.C’terminus (1:1000, Lynn Cooley—Yale University, USA), rabbit-anti-Boule (1:1000, Steven Wasserman—University of California San Diego, USA), guinea pig-anti-Tj (1:2500, Dorothea Godt—University of Toronto, Canada), rabbit-anti-Zfh1 (1:1000, Ruth Lehmann—New York University, USA), guinea pig-anti-Zfh1 (1:1000, James Skeath—Washington University in St Louis, USA), rabbit-anti-STAT (1:1000, Erika Bach—New York University, USA), rabbit-anti-pMad (1:1000, Ed Laufer—Columbia University, USA), rabbit-anti-Baz (1:1000, Tony Harris—University of Toronto), rabbit-anti-β3tub (1:4000, Renate Renkawitz-Pohl—Philipps-Universität Marburg, Germany), chicken-anti-GFP (1:1000, ab13970—Abcam), goat-anti-Vasa (1:200, dC-13—Santa Cruz), rat-anti-dsRed (1:1000, 5f8—Chromotek), chicken-anti-LacZ (1:1000, ab13970—Abcam), mouse-anti-αSpectrin (1:100, 3A9—Developmental Studies Hybridoma Bank [DSHB]), mouse-anti-Eya (1:50, eya10H6—DSHB), mouse-anti-FasIII (1:1000, 7G10—DSHB), mouse-anti-Patched (1:100, Ptc Apa 1—DSHB), mouse-anti-Dlg1 (1:2, 4F3 anti-discs large—DSHB), mouse-anti-Cora (1:500, C566.9 and C615.16—DSHB), rat-anti-DEcad (1:20, DCAD2—DSHB). Secondary antibodies included CF405S, Alexafluor-488, Cy3, and Cy5 conjugates.

### Procedures

All flies were raised at 25°C unless otherwise stated. RNAi induction in adult males (Fig 3F and 3G) done using *α1tub*-Gal80^ts^;*tj*-Gal4,*α1tub*-Gal80^ts^/UAS-*rab5*.RNAi flies raised at 18°C and split into two cohorts 1–3 Days Post Eclosion (DPE) and raised at 18°C or 29°C for 14 days. Cyst cell clones made using c587-Gal4,UAS-RFP.nls,UAS-mGFP,*hs*-Flp;*α1tub*-Gal80,Frt40A/Frt40A;UAS-*rab5*.RNAi flies (Fig 4A and 4B) and c587-Gal4,UAS-mGFP,*hs*-Flp;*α1tub*-Gal80,Frt40A/Frt40A flies (S4 Fig) raised at 20°C. Clones induced 1–5 DPE using three 45-minute heat-shocks at 37°C, each separated by 90 minutes and then aged 14 days at 20°C. Hub size (Fig 4C–4F) measured as the maximum diameter of *upd*-LacZ positive cell nuclei. JAK-STAT signalling (Fig 4G–4I) measured as the fluorescent intensity ratio of each Zfh1-positive CySC nuclei within 15μm of the hub, to the average of 2–4 GSCs contacting the hub in a single image for each testis. Hedgehog signalling (Fig 4J–4L) measured as the maximum diameter of Patched staining puncta within 20μm of the hub in a single image for each testis. Cyst cell junctions (Fig 6B and 6C and S4B Fig) imaged by dissecting single spermatocysts from L3 larval testis [73] into Schneider's Drosophila Medium and allowing them to adhere to poly-lysine coated slides for 30-minutes prior to fixation and antibody staining. Polarity protein co-localization (Fig 6E and 6G) performed on full depth, *z*-projected images encompassing 250μm of the apical tip of the testis. ImageJ (NIH) and the ‘Coloc 2’ plugin were utilized for Pearson co-localization analysis. Terminal epithelium and seminal vesicle images (Fig 7H and 7I) taken from males aged 2 days without females. Permeability assay (Fig 8D and 8E) used 10kDa dextran conjugated to AlexaFluor-647 (Life Technologies) at a final concentration of 0.2μg/μl [53]. Statistical tests done with Prism (Graphpad), all student t-tests were two-tailed and applied Welch’s correction.

## Supporting information

S1 Fig Protein-protein interaction map of candidate genes. Protein-protein interaction map of genes required in the somatic cyst cells of the *Drosophila melanogaster* testis for fertility (from Fig 1F). Map created using the STRING-Database and coloured by a summary of their Gene Ontology annotations.
(TIF)

S2 Fig Knockdown of Rab5 in cyst cells leads to overgrowth of spermatogonia-like germ cells. **(A-B)** Germline development defect resulting from cyst cell specific knockdown of Rab5. Germ cells labelled by Vasa, spectrosomes and fusomes labelled by α-Spectrin, and CySCs labelled by Zfh1. **(A’,B’)** Single channel showing α-Spectrin. **(A)** GSCs in the apical tip of the testis contain small dot shaped spectrosomes. Differentiating spermatogonia remain connected by thin, branching fusomes (**Arrowhead**) while spermatocytes are connected by large, branching fusomes (**Arrow**). **(B)** Knockdown of Rab5 in cyst cells leads to overgrowth of germ cells connected by thin, branching fusomes similar to those found in spermatogonia (**Arrowhead**). Scale bars are 50μm.
(TIF)

S3 Fig Knockdown of Rab5 in cyst cells alters Hedgehog, JAK-STAT, and BMP signalling. **(A-C)** Changes in BMP signalling after Rab5 knockdown in cyst cells. CySCs labelled by Zfh1, germ cells labelled by Vasa, BMP signalling detected by phosphorylated-Mad (pMad). Males aged 14 days post eclosion (DPE). **(A’,B’,C’)** Single channel showing pMad. **(A)** In control testes pMad is detectable in GSCs indicating active BMP signalling (**Arrowhead**). **(B)** Knockdown of Rab5 in cyst cells leads to increased levels of pMad in the germ cells near an enlarged stem cell niche (**Arrowhead**). **(C)** Increased levels of pMad are also found in the germ cell tumour-like growths that develop after knockdown of Rab5 in cyst cells. **(D)** Hh signalling is detected in the cyst cell tumour-like growths that develop outside of the stem cell niche after knockdown of Rab5. CySCs labelled by Zfh1, hub cells labelled by the Hh ligand reporter *hh*-LacZ, Hh signalling detected by Patched accumulation in CySCs. Males aged 14 DPE. **(D’)** Single channel showing Patched. **(E)** JAK-STAT signalling is detected in the cyst cell tumour-like growths that develop outside of the stem cell niche after knockdown of Rab5. CySCs labelled by Zfh1, hub cells labelled by the JAK-STAT ligand reporter *upd*-LacZ, JAK-STAT signalling detected by STAT expression in CySCs. Males aged 14 DPE. **(E’)** Single channel showing STAT. Scale bars are 20μm.
(TIF)

S4 Fig The polarity proteins Baz and Dlg1 localize to the junctional belt connecting encapsulating cyst cells. **(A)** A single cyst cell clone at the early spermatocyte-stage labelled by membrane bound GFP (mGFP). The Par polarity module protein Baz and the Scribble polarity module protein Dlg1 both localize to the membrane at the junction between the two encapsulating cyst cells (**Arrowheads**). The labelled cyst cell clone has a thin membranous extension that spreads along the sheath of the testis. **(A’)** Single channel showing Baz. **(A”)** Single channel showing Dlg1. **(B)** Individual spermatocyst extracted from the testis and labelled for the Par polarity module protein Baz::GFP and the Scribble polarity module protein Dlg1. Both proteins extend around the entire circumference of the junctional belt connecting the two encapsulating cyst cells. A section of this image appears in Fig 6C. **(B’)** Single channel showing Baz::GFP. **(B”)** Single channel showing Dlg1. Scale bars are 50μm.
(TIF)

S1 Table List of candidate genes. List of genes required in the somatic cyst cells of the *Drosophila melanogaster* testis for fertility. List includes gene names and identification, a summary of their Gene Ontology annotations, the phenotype when knocked-down in somatic cyst cells using RNAi, and mouse homologs with stage specific expression in mouse Sertoli cells (**Asterisk**) [35].
(PDF)

S2 Table Genetic screen data. This Excel file contains a number of individual sheets as follows: **(RNAi)** Is a list of all UAS-RNAi lines that were expressed in the cyst cells of the testis using tj-Gal4 in our screen as well as the raw data from the male fertility assays that were carried out subsequently. **(Genes)** Is a list of all genes targeted by RNAi knockdown in the cyst cells and a summary of the results of male fertility assays. Additional data includes gene classification, knockdown phenotype, and a comparison to prior gene annotations including the Gene Ontology (GO) term ‘spermatogenesis’, male sterile alleles listed in Flybase, phenotypes identified by other genetic screens in the somatic cells of the fly the gonad, and mouse homologs expressed in a stage specific manner in Sertoli cells. **(Sterile genes)** Is a list of candidate genes required in the somatic cyst cells of the testis for fertility (see also S1 Table). **(GOterm enrichment)** Is a list of enriched GO terms associated with candidate genes in each phenotypic class characterized using the DAVID algorithm [38]. **(Screen comparison)** Is a list comparing the results of our screen to prior screens in the somatic cells of the fly gonad including screens using *tj*-Gal4>UAS-RNAi in male fly cyst cells [31]; c587-Gal4>UAS-RNAi in male fly cyst cells [32]; and *tj*-Gal4>UAS-RNAi in female fly follicle cells [33].
(XLSX)
