# Supplementary figures and images for: Identification of genetic networks that act in the somatic cells of the testis to mediate the developmental program of spermatogenesis
Source: PLoS Genet. 2017 Sep 28;13(9):e1007026. doi: 10.1371/journal.pgen.1007026 (PMC5634645; doi:10.1371/journal.pgen.1007026)

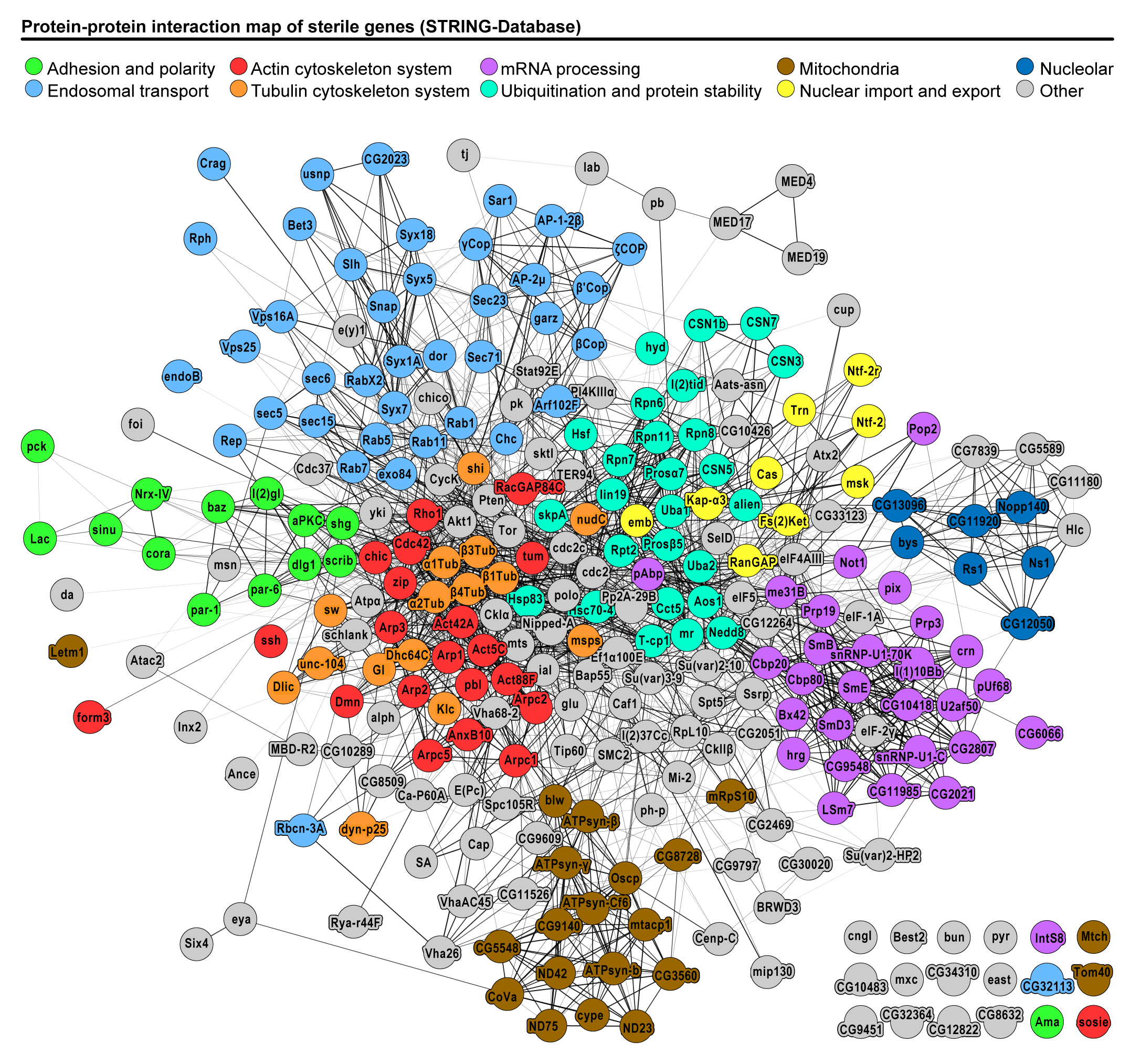

Supplement: S1 Fig — Protein-protein interaction map of genes required in the somatic cyst cells of the Drosophila melanogaster testis for fertility (from Fig 1F). Map created using the STRING-Database and coloured by a summary of their Gene Ontology annotations. (TIF) [file pgen.1007026.s001.tif]

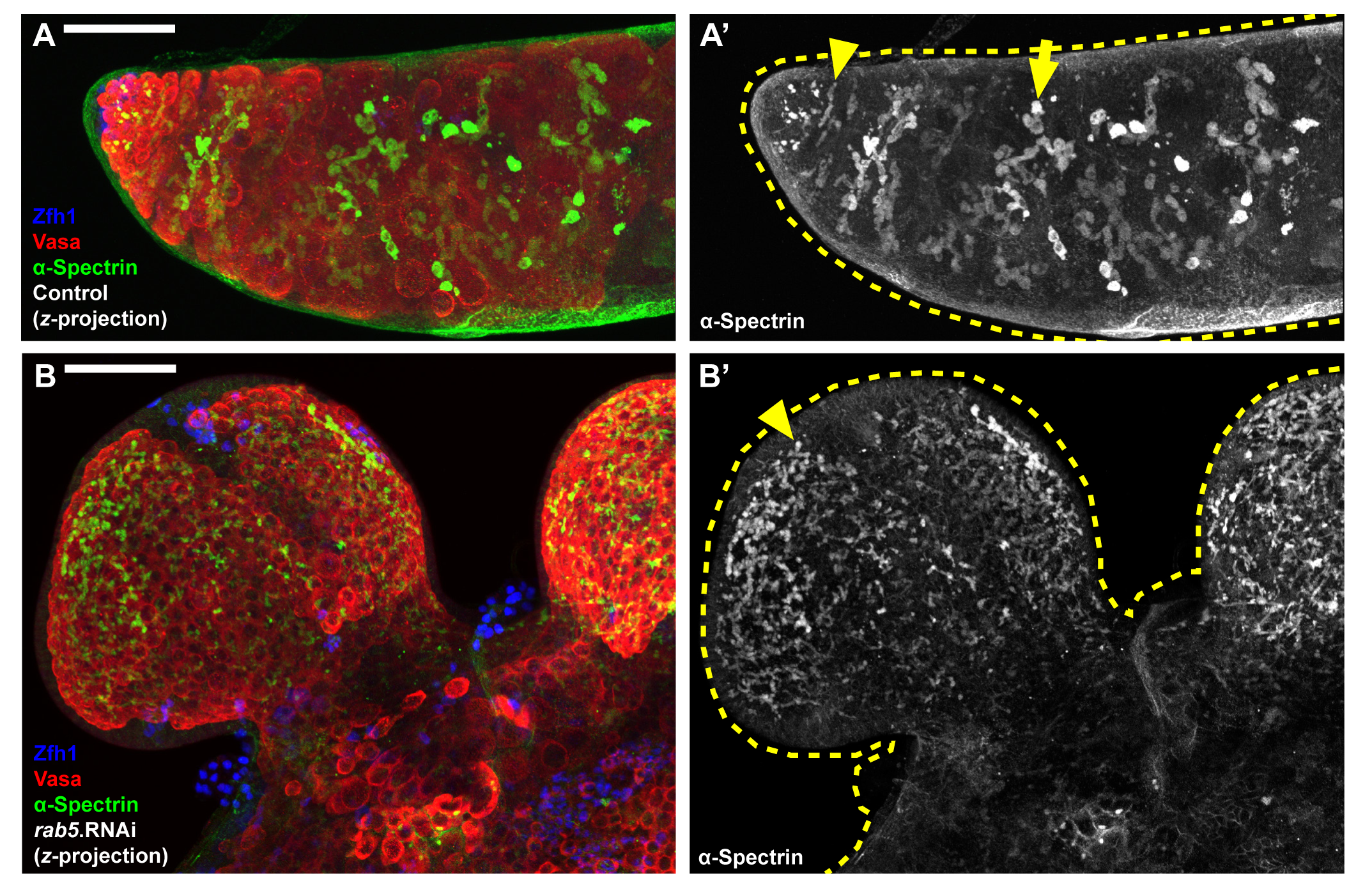

Supplement: S2 Fig — (A-B) Germline development defect resulting from cyst cell specific knockdown of Rab5. Germ cells labelled by Vasa, spectrosomes and fusomes labelled by α-Spectrin, and CySCs labelled by Zfh1. (A’,B’) Single channel showing α-Spectrin. (A) GSCs in the apical tip of the testis contain small dot shaped spectrosomes. Differentiating spermatogonia remain connected by thin, branching fusomes (Arrowhead) while spermatocytes are connected by large, branching fusomes (Arrow). (B) Knockdown of Rab5 in cyst cells leads to overgrowth of germ cells connected by thin, branching fusomes similar to those found in spermatogonia (Arrowhead). Scale bars are 50μm. (TIF) [file pgen.1007026.s002.tif]

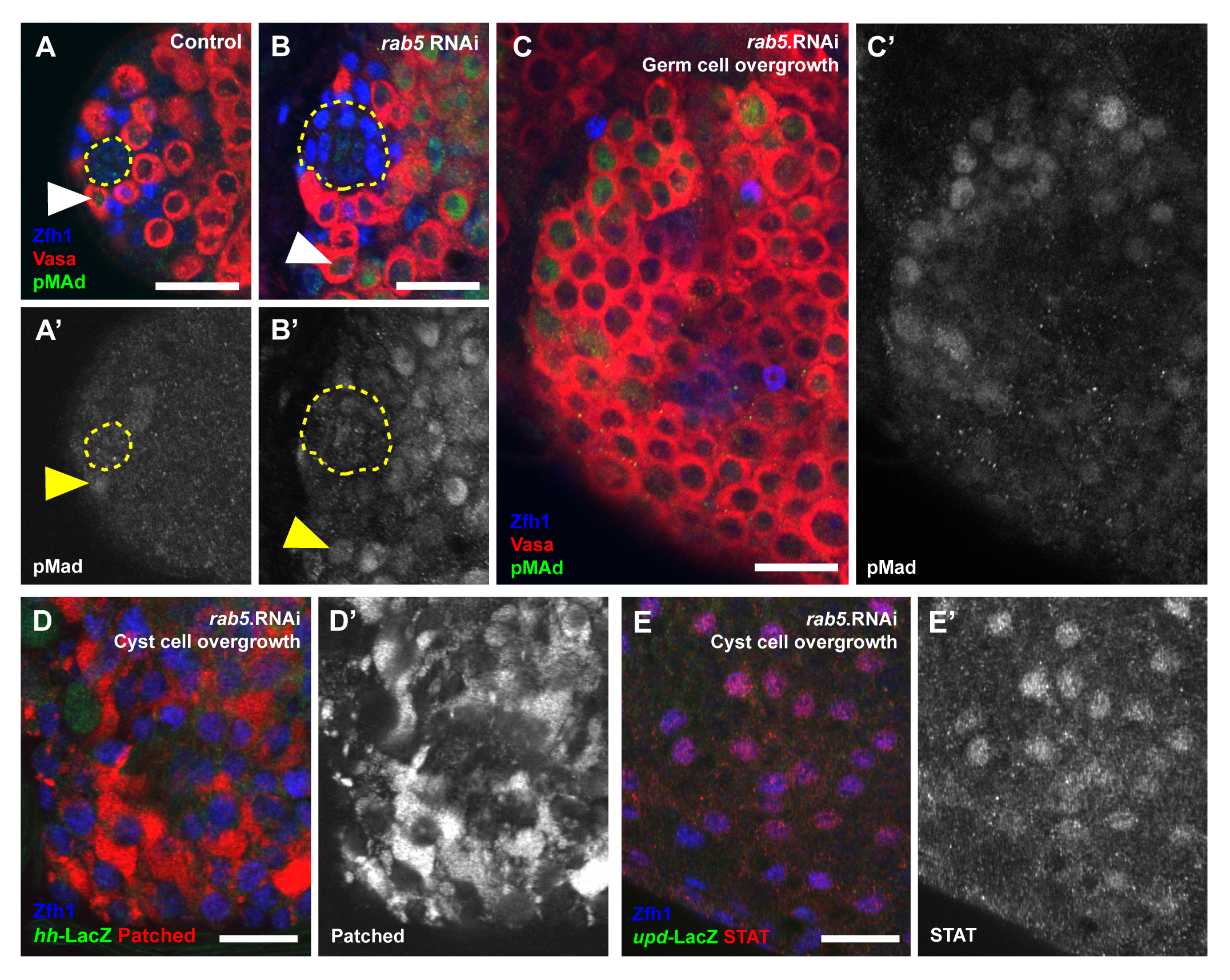

Supplement: S3 Fig — (A-C) Changes in BMP signalling after Rab5 knockdown in cyst cells. CySCs labelled by Zfh1, germ cells labelled by Vasa, BMP signalling detected by phosphorylated-Mad (pMad). Males aged 14 days post eclosion (DPE). (A’,B’,C’) Single channel showing pMad. (A) In control testes pMad is detectable in GSCs indicating active BMP signalling (Arrowhead). (B) Knockdown of Rab5 in cyst cells leads to increased levels of pMad in the germ cells near an enlarged stem cell niche (Arrowhead). (C) Increased levels of pMad are also found in the germ cell tumour-like growths that develop after knockdown of Rab5 in cyst cells. (D) Hh signalling is detected in the cyst cell tumour-like growths that develop outside of the stem cell niche after knockdown of Rab5. CySCs labelled by Zfh1, hub cells labelled by the Hh ligand reporter hh-LacZ, Hh signalling detected by Patched accumulation in CySCs. Males aged 14 DPE. (D’) Single channel showing Patched. (E) JAK-STAT signalling is detected in the cyst cell tumour-like growths that develop outside of the stem cell niche after knockdown of Rab5. CySCs labelled by Zfh1, hub cells labelled by the JAK-STAT ligand reporter upd-LacZ, JAK-STAT signalling detected by STAT expression in CySCs. Males aged 14 DPE. (E’) Single channel showing STAT. Scale bars are 20μm. (TIF) [file pgen.1007026.s003.tif]

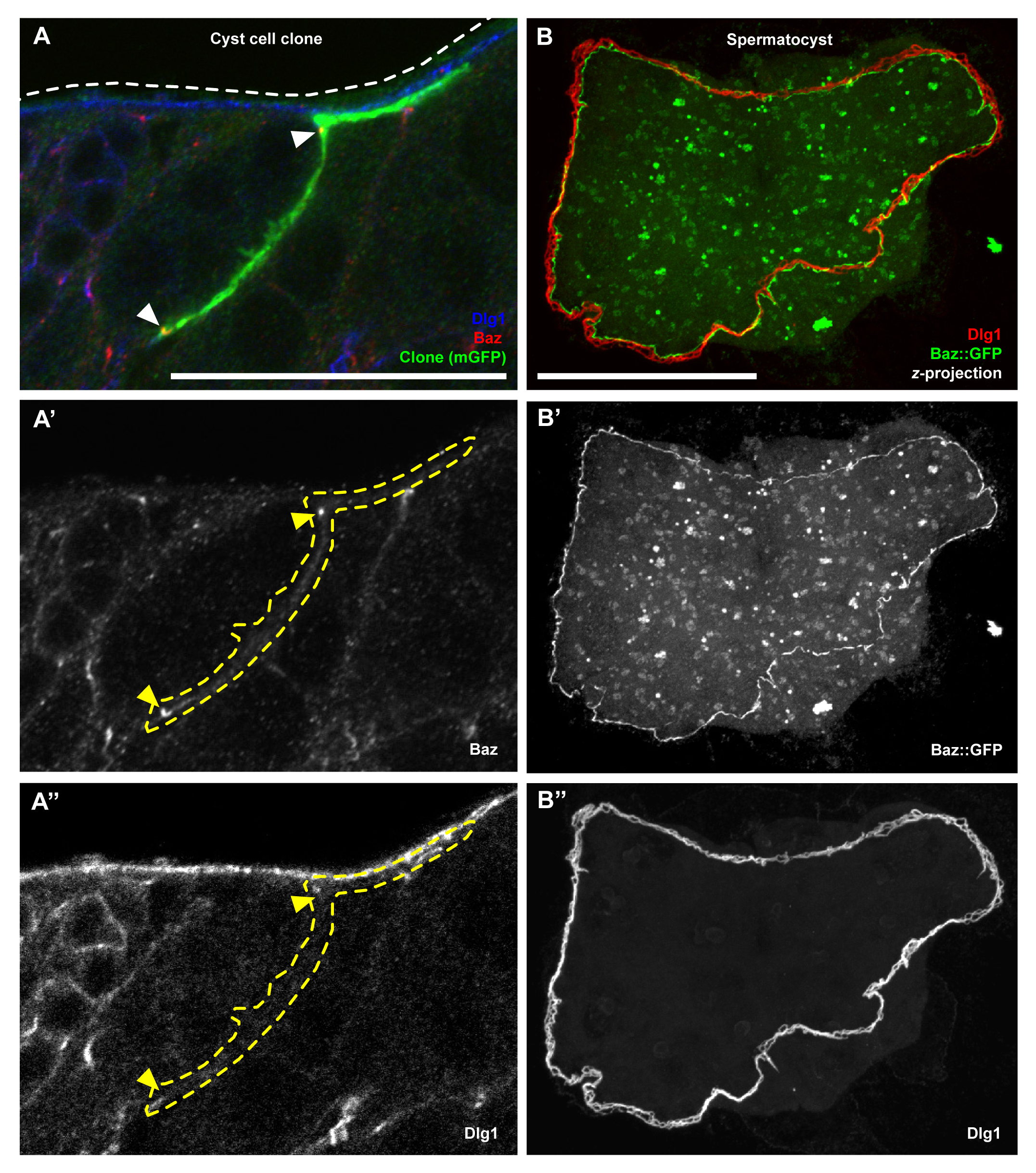

Supplement: S4 Fig — (A) A single cyst cell clone at the early spermatocyte-stage labelled by membrane bound GFP (mGFP). The Par polarity module protein Baz and the Scribble polarity module protein Dlg1 both localize to the membrane at the junction between the two encapsulating cyst cells (Arrowheads). The labelled cyst cell clone has a thin membranous extension that spreads along the sheath of the testis. (A’) Single channel showing Baz. (A”) Single channel showing Dlg1. (B) Individual spermatocyst extracted from the testis and labelled for the Par polarity module protein Baz::GFP and the Scribble polarity module protein Dlg1. Both proteins extend around the entire circumference of the junctional belt connecting the two encapsulating cyst cells. A section of this image appears in Fig 6C. (B’) Single channel showing Baz::GFP. (B”) Single channel showing Dlg1. Scale bars are 50μm. (TIF) [file pgen.1007026.s004.tif]
