# Supplementary material for: Identification of genetic networks that act in the somatic cells of the testis to mediate the developmental program of spermatogenesis
Source: PLoS Genet. 2017 Sep 28;13(9):e1007026. doi: 10.1371/journal.pgen.1007026 (PMC5634645; doi:10.1371/journal.pgen.1007026)
Supplement: S1 Table — List of genes required in the somatic cyst cells of the Drosophila melanogaster testis for fertility. List includes gene names and identification, a summary of their Gene Ontology annotations, the phenotype when knocked-down in somatic cyst cells using RNAi, and mouse homologs with stage specific expression in mouse Sertoli cells (Asterisk) [35]. (PDF) [file pgen.1007026.s005.pdf]

#	Male Sterile (*tj*>RNAi)	Gene ID	Gene Classification (Major groupings)	Testis Phenotype (tj>RNAi)	*Regulated in Sertoli cells**
1	**AP-1-2β**	CG12532	*Endomembrane Transport*	**Sp.gonial defect**	-
2	**Chc**	CG9012	*Endomembrane Transport*	**Sp.gonial defect**	-
3	**Rab5**	CG3664	*Endomembrane Transport*	**Sp.gonial defect**	-
4	**sec15**	CG7034	*Endomembrane Transport*	**Sp.gonial defect**	-
5	**Vps16A**	CG8454	*Endomembrane Transport*	**Sp.gonial defect**	-
6	**cora**	CG11949	*Adhesion and Cell Polarity*	**Sp.gonial defect**	*Epb41l3*
7	**dlg1**	CG1725	*Adhesion and Cell Polarity*	**Sp.gonial defect**	*Dlg1*
8	**l(2)gl**	CG2671	*Adhesion and Cell Polarity*	**Sp.gonial defect**	-
9	**Lac**	CG12369	*Adhesion and Cell Polarity*	**Sp.gonial defect**	*Negr1*
10	**Nrx-IV**	CG6827	*Adhesion and Cell Polarity*	**Sp.gonial defect**	*Cntnap5b*
11	**scrib**	CG43398	*Adhesion and Cell Polarity*	**Sp.gonial defect**	-
12	**Cdc42**	CG12530	*Actin Cytoskeleton*	**Sp.gonial defect**	*Rhoq*
13	**ssh**	CG6238	*Actin Cytoskeleton*	**Sp.gonial defect**	*Ssh1*
14	**alien**	CG9556	*Ubiquitination/Protein Stability*	**Sp.gonial defect**	-
15	**CSN1b**	CG3889	*Ubiquitination/Protein Stability*	**Sp.gonial defect**	-
16	**CSN3**	CG18332	*Ubiquitination/Protein Stability*	**Sp.gonial defect**	-
17	**Kap-α3**	CG9423	*Nuclear Import and Export*	**Sp.gonial defect**	*Kpna6*
18	**Atx2**	CG5166	*mRNA Regulation*	**Sp.gonial defect**	-
19	**IntS8**	CG5859	*mRNA Regulation*	**Sp.gonial defect**	-
20	**LSm7**	CG13277	*mRNA Regulation*	**Sp.gonial defect**	-
21	**pix**	CG5651	*mRNA Regulation*	**Sp.gonial defect**	-
22	**Pop2**	CG5684	*mRNA Regulation*	**Sp.gonial defect**	*Cnot7*
23	**snRNP-U1-70K**	CG8749	*mRNA Regulation*	**Sp.gonial defect**	-
24	**U2af50**	CG9998	*mRNA Regulation*	**Sp.gonial defect**	-
25	**CG6066**	CG6066	*mRNA Regulation*	**Sp.gonial defect**	-
26	**ATPsynB**	CG8189	*Mitochondrial*	**Sp.gonial defect**	-
27	**ATPsynγ**	CG7610	*Mitochondrial*	**Sp.gonial defect**	-
28	**Mtch**	CG6851	*Mitochondrial*	**Sp.gonial defect**	-
29	**Tom40**	CG12157	*Mitochondrial*	**Sp.gonial defect**	-
30	**Atpα**	CG5670	-	**Sp.gonial defect**	*Atp1a1*
31	**cngl**	CG43395	-	**Sp.gonial defect**	-
32	**MED19**	CG5546	-	**Sp.gonial defect**	-
33	**MED4**	CG8609	-	**Sp.gonial defect**	-
34	**chico**	CG5686	-	**Sp.gonial defect**	-
35	**E(Pc)**	CG7776	-	**Sp.gonial defect**	*Epc1*
36	**SA**	CG3423	-	**Sp.gonial defect**	*Stag3*
37	**Tip60**	CG6121	-	**Sp.gonial defect**	-
38	**CG5589**	CG5589	-	**Sp.gonial defect**	-
39	**CG9609**	CG9609	-	**Sp.gonial defect**	-
40	**CkIIβ**	CG15224	-	**Sp.gonial defect**	-
41	**CkIα**	CG2028	-	**Sp.gonial defect**	*Csnk1e*
42	**mts**	CG7109	-	**Sp.gonial defect**	-

43	**AP-2μ**	CG7057	*Endomembrane Transport*	**Sp.cyte defect**	-
44	**Syx1A**	CG31136	*Endomembrane Transport*	**Sp.cyte defect**	-
45	**Syx7**	CG5081	*Endomembrane Transport*	**Sp.cyte defect**	-
46	**aPKC**	CG42783	*Adhesion and Cell Polarity*	**Sp.cyte defect**	-
47	**baz**	CG5055	*Adhesion and Cell Polarity*	**Sp.cyte defect**	*Pard3*
48	**par-1**	CG8201	*Adhesion and Cell Polarity*	**Sp.cyte defect**	*Mark2*
49	**par-6**	CG5884	*Adhesion and Cell Polarity*	**Sp.cyte defect**	*Pard6g*
50	**pck**	CG14779	*Adhesion and Cell Polarity*	**Sp.cyte defect**	-
51	**sinu**	CG10624	*Adhesion and Cell Polarity*	**Sp.cyte defect**	-
52	**Arf102F**	CG11027	*Endomembrane Transport*	**Sp.tid defect**	*Arf3*
53	**Crag**	CG12737	*Endomembrane Transport*	**Sp.tid defect**	-
54	**dor**	CG3093	*Endomembrane Transport*	**Sp.tid defect**	*Vps18*
55	**endoB**	CG9834	*Endomembrane Transport*	**Sp.tid defect**	*Sh3glb1*
56	**Rab7**	CG5915	*Endomembrane Transport*	**Sp.tid defect**	*Rab7b*
57	**Rbcn-3A**	CG3585	*Endomembrane Transport*	**Sp.tid defect**	-
58	**Rep**	CG8432	*Endomembrane Transport*	**Sp.tid defect**	*Chm*
59	**sec71**	CG7578	*Endomembrane Transport*	**Sp.tid defect**	-
60	**Snap29**	CG11173	*Endomembrane Transport*	**Sp.tid defect**	-
61	**Syx5**	CG4214	*Endomembrane Transport*	**Sp.tid defect**	*Stx5a*
62	**Vps25**	CG14750	*Endomembrane Transport*	**Sp.tid defect**	-
63	**Shg**	CG3722	*Adhesion and Cell Polarity*	**Sp.tid defect**	-
64	**Act5C**	CG4027	*Actin Cytoskeleton*	**Sp.tid defect**	*Acta2*
65	**AnxB10**	CG9579	*Actin Cytoskeleton*	**Sp.tid defect**	*Anxa6*
66	**Arp1**	CG6174	*Actin Cytoskeleton*	**Sp.tid defect**	*Acta2*
67	**Arp2**	CG9901	*Actin Cytoskeleton*	**Sp.tid defect**	-
68	**Arp3**	CG7558	*Actin Cytoskeleton*	**Sp.tid defect**	*Actr3b*
69	**Arpc1**	CG8978	*Actin Cytoskeleton*	**Sp.tid defect**	-
70	**Arpc2**	CG10954	*Actin Cytoskeleton*	**Sp.tid defect**	-
71	**Arpc5**	CG9881	*Actin Cytoskeleton*	**Sp.tid defect**	*Arpc5*
72	**Dmn**	CG8269	*Actin Cytoskeleton*	**Sp.tid defect**	-
73	**RacGAP84C**	CG2595	*Actin Cytoskeleton*	**Sp.tid defect**	*Racgap1*
74	**zip**	CG15792	*Actin Cytoskeleton*	**Sp.tid defect**	*Myh10*
75	**Dhc64C**	CG7507	*Tubulin Cytoskeleton*	**Sp.tid defect**	*Dnah8*
76	**Dlic**	CG1938	*Tubulin Cytoskeleton*	**Sp.tid defect**	*Dync1li2*
77	**dyn-p25**	CG10846	*Tubulin Cytoskeleton*	**Sp.tid defect**	*Dctn5*
78	**Gl**	CG9206	*Tubulin Cytoskeleton*	**Sp.tid defect**	*Dctn1*
79	**msps**	CG5000	*Tubulin Cytoskeleton*	**Sp.tid defect**	*Ckap5*
80	**sw**	CG18000	*Tubulin Cytoskeleton*	**Sp.tid defect**	-
81	**α1Tub**	CG1913	*Tubulin Cytoskeleton*	**Sp.tid defect**	*Tuba3b*
82	**α2Tub**	CG9476	*Tubulin Cytoskeleton*	**Sp.tid defect**	*Tuba3a*
83	**β3Tub**	CG3401	*Tubulin Cytoskeleton*	**Sp.tid defect**	*Tubb2b*
84	**β4Tub**	CG4869	*Tubulin Cytoskeleton*	**Sp.tid defect**	*Tubb2a*
85	**Hsf**	CG5748	*Ubiquitination/Protein Stability*	**Sp.tid defect**	-
86	**Hsp83**	CG1242	*Ubiquitination/Protein Stability*	**Sp.tid defect**	*Hsp90b1*

87	**l(2)tid**	CG5504	*Ubiquitination/Protein Stability*	**Sp.tid defect**	-
88	**Trn**	CG7398	*Nuclear Import and Export*	**Sp.tid defect**	*Tnpo2*
89	**Nopp140**	CG7421	*Nucleolar*	**Sp.tid defect**	-
90	**ns1**	CG3983	*Nucleolar*	**Sp.tid defect**	-
91	**me31B**	CG4916	*mRNA Regulation*	**Sp.tid defect**	-
92	**CG2021**	CG2021	*mRNA Regulation*	**Sp.tid defect**	-
93	**blw**	CG3612	*Mitochondrial*	**Sp.tid defect**	-
94	**COX5A**	CG14724	*Mitochondrial*	**Sp.tid defect**	*Cox5a*
95	**cype**	CG14028	*Mitochondrial*	**Sp.tid defect**	*Cox6c*
96	**mRpS10**	CG4247	*Mitochondrial*	**Sp.tid defect**	-
97	**ND-23**	CG3944	*Mitochondrial*	**Sp.tid defect**	-
98	**ND-42**	CG6343	*Mitochondrial*	**Sp.tid defect**	-
99	**ND-51**	CG9140	*Mitochondrial*	**Sp.tid defect**	-
100	**ND-75**	CG2286	*Mitochondrial*	**Sp.tid defect**	-
101	**ND-B18**	CG5548	*Mitochondrial*	**Sp.tid defect**	-
102	**ZnT49B**	CG8632	-	**Sp.tid defect**	-
103	**da**	CG5102	-	**Sp.tid defect**	*Tcf12*
104	**yki**	CG4005	-	**Sp.tid defect**	*Yap1*
105	**bun**	CG42281	-	**Sp.tid defect**	*Tsc22d1*
106	**msn**	CG16973	-	**Sp.tid defect**	*Tnik*
107	**Pten**	CG5671	-	**Sp.tid defect**	-
108	**pyr**	CG13194	-	**Sp.tid defect**	-
109	**Hlc**	CG1666	-	**Sp.tid defect**	-
110	**PI4KIIIα**	CG10260	-	**Sp.tid defect**	*Pi4ka*
111	**schlank**	CG3576	-	**Sp.tid defect**	*Cers5*
112	**sktl**	CG9985	-	**Sp.tid defect**	*Pip5k1b*
113	**TER94**	CG2331	-	**Sp.tid defect**	-
114	**Bet3**	CG3911	*Endomembrane Transport*	Cyst cells absent	-
115	**eIF-1A**	CG8053	*Endomembrane Transport*	Cyst cells absent	-
116	**exo84**	CG6095	*Endomembrane Transport*	Cyst cells absent	-
117	**Rab1**	CG3320	*Endomembrane Transport*	Cyst cells absent	-
118	**Rab11**	CG5771	*Endomembrane Transport*	Cyst cells absent	*Rab11a*
119	**Rph**	CG11556	*Endomembrane Transport*	Cyst cells absent	*Doc2b*
120	**sar1**	CG7073	*Endomembrane Transport*	Cyst cells absent	-
121	**sec23**	CG1250	*Endomembrane Transport*	Cyst cells absent	-
122	**sec5**	CG8843	*Endomembrane Transport*	Cyst cells absent	*Exoc2*
123	**Slh**	CG3539	*Endomembrane Transport*	Cyst cells absent	-
124	**Syx18**	CG13626	*Endomembrane Transport*	Cyst cells absent	-
125	**β'Cop**	CG6699	*Endomembrane Transport*	Cyst cells absent	-
126	**βCop**	CG6223	*Endomembrane Transport*	Cyst cells absent	*Copb1*
127	**γCop**	CG1528	*Endomembrane Transport*	Cyst cells absent	-
128	**ζCOP**	CG3948	*Endomembrane Transport*	Cyst cells absent	*Copz1*
129	**CG2023**	CG2023	*Endomembrane Transport*	Cyst cells absent	-
130	**CG32113**	CG32113	*Endomembrane Transport*	Cyst cells absent	-
131	**Ama**	CG2198	*Adhesion and Cell Polarity*	Cyst cells absent	-

132	**pk**	CG11084	*Adhesion and Cell Polarity*	Cyst cells absent	*Fhl5*
133	**Act88F**	CG5178	*Actin Cytoskeleton*	Cyst cells absent	*Acta2*
134	**chic**	CG9553	*Actin Cytoskeleton*	Cyst cells absent	-
135	**form3**	CG33556	*Actin Cytoskeleton*	Cyst cells absent	*Daam2*
136	**pbl**	CG8114	*Actin Cytoskeleton*	Cyst cells absent	*Ect2*
137	**Rho1**	CG8416	*Actin Cytoskeleton*	Cyst cells absent	*Rhoc*
138	**tum**	CG13345	*Actin Cytoskeleton*	Cyst cells absent	*Racgap1*
139	**nudC**	CG9710	*Tubulin Cytoskeleton*	Cyst cells absent	*Nudc*
140	**shi**	CG18102	*Tubulin Cytoskeleton*	Cyst cells absent	*Dnm3*
141	**unc-104**	CG8566	*Tubulin Cytoskeleton*	Cyst cells absent	*Kif1c*
142	**Cct5**	CG8439	*Ubiquitination/Protein Stability*	Cyst cells absent	-
143	**Hsc70-4**	CG4264	*Ubiquitination/Protein Stability*	Cyst cells absent	*Hspa2*
144	**hyd**	CG9484	*Ubiquitination/Protein Stability*	Cyst cells absent	-
145	**Prosα7**	CG1519	*Ubiquitination/Protein Stability*	Cyst cells absent	-
146	**Prosβ5**	CG12323	*Ubiquitination/Protein Stability*	Cyst cells absent	-
147	**Rpn11**	CG18174	*Ubiquitination/Protein Stability*	Cyst cells absent	-
148	**Rpn6**	CG10149	*Ubiquitination/Protein Stability*	Cyst cells absent	-
149	**Rpn7**	CG5378	*Ubiquitination/Protein Stability*	Cyst cells absent	-
150	**Rpn8**	CG3416	*Ubiquitination/Protein Stability*	Cyst cells absent	*Psmd7*
151	**Rpt2**	CG5289	*Ubiquitination/Protein Stability*	Cyst cells absent	-
152	**skpA**	CG16983	*Ubiquitination/Protein Stability*	Cyst cells absent	-
153	**T-cp1**	CG5374	*Ubiquitination/Protein Stability*	Cyst cells absent	-
154	**Uba1**	CG1782	*Ubiquitination/Protein Stability*	Cyst cells absent	*Uba1y*
155	**Uba2**	CG7528	*Ubiquitination/Protein Stability*	Cyst cells absent	-
156	**Cas**	CG13281	*Nuclear Import and Export*	Cyst cells absent	-
157	**emb**	CG13387	*Nuclear Import and Export*	Cyst cells absent	*Xpo1*
158	**Fs(2)Ket**	CG2637	*Nuclear Import and Export*	Cyst cells absent	-
159	**msk**	CG7935	*Nuclear Import and Export*	Cyst cells absent	-
160	**Ntf-2**	CG1740	*Nuclear Import and Export*	Cyst cells absent	-
161	**RanGAP**	CG9999	*Nuclear Import and Export*	Cyst cells absent	-
162	**bys**	CG1430	*Nucleolar*	Cyst cells absent	-
163	**l(2)05287**	CG12050	*Nucleolar*	Cyst cells absent	-
164	**Rs1**	CG2173	*Nucleolar*	Cyst cells absent	-
165	**CG11920**	CG11920	*Nucleolar*	Cyst cells absent	-
166	**CG13096**	CG13096	*Nucleolar*	Cyst cells absent	-
167	**Bx42**	CG8264	*mRNA Regulation*	Cyst cells absent	-
168	**Cbp20**	CG12357	*mRNA Regulation*	Cyst cells absent	-
169	**Cbp80**	CG7035	*mRNA Regulation*	Cyst cells absent	-
170	**crn**	CG3193	*mRNA Regulation*	Cyst cells absent	-
171	**hfp**	CG12085	*mRNA Regulation*	Cyst cells absent	-
172	**hrg**	CG9854	*mRNA Regulation*	Cyst cells absent	*Papola*
173	**l(1)10Bb**	CG1639	*mRNA Regulation*	Cyst cells absent	-
174	**Not1**	CG34407	*mRNA Regulation*	Cyst cells absent	*Cnot1*
175	**pAbp**	CG5119	*mRNA Regulation*	Cyst cells absent	*Pabpc4*
176	**Prp19**	CG5519	*mRNA Regulation*	Cyst cells absent	-
177	**Prp3**	CG7757	*mRNA Regulation*	Cyst cells absent	-

178	**SmB**	CG5352	*mRNA Regulation*	Cyst cells absent	-
179	**SmD3**	CG8427	*mRNA Regulation*	Cyst cells absent	-
180	**SmE**	CG18591	*mRNA Regulation*	Cyst cells absent	-
181	**snRNP-U1-C**	CG5454	*mRNA Regulation*	Cyst cells absent	-
182	**CG10418**	CG10418	*mRNA Regulation*	Cyst cells absent	-
183	**CG11985**	CG11985	*mRNA Regulation*	Cyst cells absent	-
184	**CG2807**	CG2807	*mRNA Regulation*	Cyst cells absent	-
185	**CG9548**	CG9548	*mRNA Regulation*	Cyst cells absent	-
186	**ATPsynCF6**	CG4412	*Mitochondrial*	Cyst cells absent	-
187	**ATPsynO**	CG4307	*Mitochondrial*	Cyst cells absent	-
188	**ATPsynβ**	CG11154	*Mitochondrial*	Cyst cells absent	-
189	**Letm1**	CG4589	*Mitochondrial*	Cyst cells absent	*Letm2*
190	**SERCA**	CG3725	-	Cyst cells absent	*Atp2a2*
191	**Vha26**	CG1088	-	Cyst cells absent	*Atp6v1e1*
192	**AsnRS**	CG10687	-	Cyst cells absent	-
193	**eIF-2γ**	CG43665	-	Cyst cells absent	*Suv39h2*
194	**eIF4AIII**	CG7483	-	Cyst cells absent	-
195	**eIF5**	CG9177	-	Cyst cells absent	-
196	**LeuRS**	CG33123	-	Cyst cells absent	*Lars*
197	**e(y)1**	CG6474	-	Cyst cells absent	-
198	**eya**	CG9554	-	Cyst cells absent	-
199	**lab**	CG1264	-	Cyst cells absent	-
200	**Spt5**	CG7626	-	Cyst cells absent	*Supt5*
201	**Stat92E**	CG4257	-	Cyst cells absent	*Stat5b*
202	**Akt1**	CG4006	-	Cyst cells absent	*Sgk1*
203	**alph**	CG1906	-	Cyst cells absent	-
204	**Ance**	CG8827	-	Cyst cells absent	*Ace*
205	**Strip**	CG11526	-	Cyst cells absent	*Strip2*
206	**Tor**	CG5092	-	Cyst cells absent	*Mtor*
207	**CG10483**	CG10483	-	Cyst cells absent	-
208	**Atac2**	CG10414	-	Cyst cells absent	-
209	**aurB**	CG6620	-	Cyst cells absent	*Aurkb*
210	**BRWD3**	CG31132	-	Cyst cells absent	-
211	**Caf1-55**	CG4236	-	Cyst cells absent	-
212	**Cdk1**	CG5363	-	Cyst cells absent	*Cdk1*
213	**Cenp-C**	CG31258	-	Cyst cells absent	-
214	**CycK**	CG15218	-	Cyst cells absent	*Ccnk*
215	**east**	CG4399	-	Cyst cells absent	-
216	**glu**	CG11397	-	Cyst cells absent	*Smc4*
217	**Hat1**	CG2051	-	Cyst cells absent	*Hat1*
218	**MBD-R2**	CG10042	-	Cyst cells absent	*Kmt2e*
219	**Mi-2**	CG8103	-	Cyst cells absent	*Chd3*
220	**mxc**	CG12124	-	Cyst cells absent	-
221	**Nipped-A**	CG33554	-	Cyst cells absent	*Trrap*
222	**SMC2**	CG10212	-	Cyst cells absent	*Smc2*
223	**SMC3**	CG9802	-	Cyst cells absent	-

224	**Spc105R**	CG11451	-	Cyst cells absent	-
225	**Ssrp**	CG4817	-	Cyst cells absent	-
226	**Su(var)2-10**	CG8068	-	Cyst cells absent	*Zmiz1*
227	**Su(var)2-HP2**	CG12864	-	Cyst cells absent	*Rif1*
228	**Su(var)3-9**	CG43664	-	Cyst cells absent	*Suv39h2*
229	**CG2469**	CG2469	-	Cyst cells absent	*Ctr9*
230	**INPP5E**	CG10426	-	Cyst cells absent	-
231	**Cdc37**	CG12019	-	Cyst cells absent	*Cdc37l1*
232	**l(2)37Cc**	CG10691	-	Cyst cells absent	-
233	**M1BP**	CG9797	-	Cyst cells absent	*Zfp24*
234	**Pp2A-29B**	CG17291	-	Cyst cells absent	-
235	**SelD**	CG8553	-	Cyst cells absent	*Sephs1*
236	**tut**	CG32364	-	Cyst cells absent	-
237	**CG11180**	CG11180	-	Cyst cells absent	-
238	**CG12264**	CG12264	-	Cyst cells absent	-
239	**CG7839**	CG7839	-	Cyst cells absent	-
240	**garz**	CG8487	*Endomembrane Transport*	**-**	*Gbf1*
241	**RabX2**	CG2885	*Endomembrane Transport*	**-**	-
242	**sec6**	CG5341	*Endomembrane Transport*	**-**	*Exoc3l*
243	**αSnap**	CG6625	*Endomembrane Transport*	**-**	-
244	**Act42A**	CG12051	*Actin Cytoskeleton*	**-**	*Acta2*
245	**sosie**	CG13636	*Actin Cytoskeleton*	**-**	-
246	**Klc**	CG5433	*Tubulin Cytoskeleton*	**-**	*Klc4*
247	**β1Tub**	CG9277	*Tubulin Cytoskeleton*	**-**	*Tubb2b*
248	**Aos1**	CG12276	*Ubiquitination/Protein Stability*	**-**	-
249	**CSN5**	CG14884	*Ubiquitination/Protein Stability*	**-**	-
250	**CSN7**	CG2038	*Ubiquitination/Protein Stability*	**-**	-
251	**Cul1**	CG1877	*Ubiquitination/Protein Stability*	**-**	-
252	**mr**	CG3060	*Ubiquitination/Protein Stability*	**-**	-
253	**Nedd8**	CG10679	*Ubiquitination/Protein Stability*	**-**	-
254	**Ntf-2r**	CG10174	*Nuclear Import and Export*	**-**	-
255	**ND-ACP**	CG9160	*Mitochondrial*	**-**	-
256	**UQCR-14**	CG3560	*Mitochondrial*	**-**	-
257	**CG8728**	CG8728	*Mitochondrial*	**-**	-
258	**Bap55**	CG6546	-	**-**	*Actl6a*
259	**Best2**	CG10173	-	**-**	*Best1*
260	**foi**	CG6817	-	**-**	*Slc39a10*
261	**inx2**	CG4590	-	**-**	-
262	**RyR**	CG10844	-	**-**	-
263	**Vha68-2**	CG3762	-	**-**	-
264	**VhaAC45**	CG8029	-	**-**	-
265	**Ef1α100E**	CG1873	-	**-**	-
266	**RpL10**	CG17521	-	**-**	-
267	**MED17**	CG7957	-	**-**	-
268	**pb**	CG31481	-	**-**	-

269	**Six4**	CG3871	-	-	*Six5*
270	**tj**	CG10034	-	-	-
271	**Cdk2**	CG10498	-	-	*Cdk1*
272	**mip130**	CG3480	-	-	-
273	**ph-p**	CG18412	-	-	*Phc3*
274	**polo**	CG12306	-	-	*Plk1*
275	**CG30020**	CG30020	-	-	*Zfp580*
276	**cup**	CG11181	-	-	-
277	**CG10289**	CG10289	-	-	*Ppp6r2*
278	**CG12822**	CG12822	-	-	-
279	**CG34310**	CG34310	-	-	-
280	**CG8509**	CG8509	-	-	-
281	**CG9451**	CG9451	-	-	-
